# Arabidopsis MEB3 functions as a vacuolar metal transporter to regulate iron accumulation in roots

**DOI:** 10.3389/fpls.2025.1517144

**Published:** 2025-03-06

**Authors:** Kaichiro Endo, Arpan Kumar Basak, Alwine Wilkens, Mohamadreza Mirzaei, Stanislav Kopriva, Kenji Yamada

**Affiliations:** ^1^ Malopolska Centre of Biotechnology, Jagiellonian University, Krakow, Poland; ^2^ Faculty of Biology, Jagiellonian University, Krakow, Poland; ^3^ The Franciszek Gorski Institute of Plant Physiology, Polish Academy of Sciences, Krakow, Poland; ^4^ Doctoral School of Exact and Natural Sciences, Jagiellonian University, Krakow, Poland; ^5^ Institute for Plant Sciences, Cluster of Excellence on Plant Sciences (CEPLAS), University of Cologne, Cologne, Germany

**Keywords:** Arabidopsis, transporter, iron, metal, vacuole

## Abstract

Iron is an essential nutrient for plant photosynthesis and development, but excess iron leads to stress. After absorption from the soil, plants store iron in roots and distribute it to shoots via long-distance transport. The vacuole is involved in iron storage and the maintenance of cellular iron homeostasis, and vacuolar iron transporter (VIT) family proteins have been identified as plant vacuolar iron transporters. However, the contribution of vacuolar iron transporters to overall iron homeostasis in plants is not fully understood. Here, we show that MEMBRANE PROTEIN OF ER BODY 3 (MEB3), a VIT family member, functions as a vacuolar metal transporter for iron distribution in *Arabidopsis thaliana*. Heterologous expression of Arabidopsis *MEB3* in yeast vacuolar iron or zinc transporter mutants restored the iron- and zinc-resistance phenotypes of the respective mutants, indicating that MEB3 regulates iron and zinc transport. In Arabidopsis, *MEB3* was expressed in almost all tissues, albeit to higher levels in roots and seedlings, and MEB3 protein localized to the tonoplast. Iron but not zinc levels were reduced in *meb3* knockout mutant roots, suggesting that the knockout reduced iron storage capacity in roots. At high iron concentration, *meb3* mutants accumulated more iron in shoots and less iron in roots than the wild type, indicating impairment of proper iron distribution in *meb3* mutants. These findings demonstrate that MEB3 is a vacuolar transporter involved in the homeostasis of iron and other metals in plants.

## 1 Introduction

Iron is a heavy metal essential for many processes related to plant growth (Chaffai and Koyama, 2011; Andresen et al., 2018), such as photosynthesis (Jeong and Guerinot, 2009; Kroh and Pilon, 2020), chlorophyll biosynthesis (Mochizuki et al., 2010), metabolic gene expression (López-Millán et al., 2013), and suppression of glycolysis and phloem glucose loading (Thimm et al., 2001). Therefore, the regulation of iron content and distribution in tissues and organs is deeply connected to the survival strategy of plants.

Ferric ions (Fe^3+^) in soil are reduced to ferrous ions (Fe^2+^) by FERRIC REDUCTION OXIDASE 2 (FRO2) on the surface of the root epidermis (Robinson et al., 1999; Connolly et al., 2003), and are then transported into root cells via the plasma membrane-localized IRON REGULATED TRANSPORTER 1 (IRT1) protein (Connolly et al., 2002; Vert et al., 2002). Iron uptake in roots and iron translocation to shoots are enhanced by iron deficiency (Schwarz and Bauer, 2020). The *FE-DEFICIENCY INDUCED TRANSCRIPTION FACTOR* (*FIT*) and type Ib basic helix–loop–helix (bHLH) transcription factor genes (*bHLH38*, *bHLH39*, *bHLH100*, *bHLH101*) are induced under iron-deficient conditions (Colangelo and Guerinot, 2004; Gao and Dubos, 2021), after which they form heterodimer complexes to directly regulate the expression of *FRO2* and *IRT1* to promote iron uptake. Moreover, the *AHA2* gene, which encodes a plasma membrane H^+^-ATPase, is induced in root cells under iron-deficient conditions to increase Fe^3+^ solubility via acidification of the soil (Santi and Schmidt, 2009).

In plant cells, iron is distributed to plastids and mitochondria for various applications (Andresen et al., 2018), but excess iron in these organelles is buffered by ferritin proteins or stored in vacuoles and cell walls (Lanquar et al., 2010; Nouet et al., 2011; Donner et al., 2012; Andresen et al., 2018; Liu et al., 2023). Arabidopsis VACUOLAR IRON TRANSPORTER 1 (VIT1), a homolog of yeast (*Saccharomyces cerevisiae*) CCC1, is responsible for vacuolar iron storage in the provascular and endodermal cells of the embryo, which is important for early seed germination (Kim et al., 2006; Donner et al., 2012; Grillet et al., 2014). VIT1 and its homologs possess a conserved multispanning transmembrane region, named DOMAIN OF UNKNOWN FUNCTION 125 (DUF125), and these proteins together form a large VIT family. The VIT family includes Arabidopsis VACUOLAR IRON TRANSPORTER LIKE 1 (VTL1), VTL2, and VTL5, *Lotus japonicus* SEN1, soybean (*Glycine max*) nodulin-21/GmVTL1, and Arabidopsis MEMBRANE PROTEIN OF ER BODY 1 (MEB1) and MEB2 (Yamada et al., 2013). Arabidopsis VTL1, VTL2, VTL5, MEB1, and MEB2 proteins exhibit iron transport activity when the corresponding genes are heterologously expressed in yeast (Yamada et al., 2013; Gollhofer et al., 2014). Arabidopsis *VTL1* encodes a vacuolar protein and is expressed in the roots, hypocotyls, and cotyledons of seedlings, suggesting that VTL1 is a major vacuolar iron transporter that regulates iron accumulation in seedlings (Gollhofer et al., 2011). In mature Arabidopsis plants, the expression of *VTL1* is downregulated by iron deficiency, suggesting that plants reduce the level of vacuolar iron transporters to increase the mobilization of vacuolar iron (Yan et al., 2016). In the Fabaceae family, *L. japonicus SEN1* and soybean *GmVTL1* are expressed in root nodules and are suggested to support in nitrogen fixation (Delauney et al., 1990; Hakoyama et al., 2012). Furthermore, alfalfa (*Medicago truncatula*) MtVTL8 and GmVTL1 function as iron transporters in root nodules (Brear et al., 2020; Liu et al., 2020; Walton et al., 2020).

Besides VIT family proteins, FERROPORTIN 2 (FPN2) has also been identified as a vacuolar iron transporter (Morrissey et al., 2009), while NATURAL RESISTANCE-ASSOCIATED MACROPHAGE PROTEIN 3 (NRAMP3) and NRAMP4 have been identified as vacuolar iron exporters in Arabidopsis (Lanquar et al., 2005). The expression of *NRAMP3* and *NRAMP4* is upregulated in Arabidopsis roots under iron-deficient conditions, indicating that *NRAMP3* and *NRAMP4* are responsible for iron mobilization from vacuoles during iron deficiency. Together with the VIT family proteins, these iron importers and exporters contribute to iron mobilization in plants. To enable the long-distance transport of iron from roots to shoots, the metal ions are converted into metal-chelate complexes such as Fe^3+^-citrate, Fe^3+^-mugineic acid, and Fe^2+^-nicotianamine (Fe^2+^-NA) (Kobayashi and Nishizawa, 2012).

Unlike other VIT family proteins, which localize to vacuoles, Arabidopsis MEB1 and MEB2 proteins localize to endoplasmic reticulum (ER) bodies (Yamada et al., 2013). ER bodies are ER-derived, rod-shaped organelles in the Brassicaceae plant family and closely related Cleomaceae and Capparaceae plant families (Behnke and Eschlbeck, 1978). ER bodies accumulate β-glucosidases, which hydrolyze glucosinolates, releasing metabolites that repel herbivores (Nakazaki et al., 2019; Yamada et al., 2020) and facilitate root microbiota establishment in Arabidopsis (Hara-Nishimura and Matsushima, 2003; Yamada et al., 2009; Basak et al., 2024). The function of MEB1 and MEB2 in glucosinolate metabolism remains obscure, but both proteins exhibit iron and manganese transport activity and play a role in maintaining the rod-shaped morphology of ER bodies (Yamada et al., 2013; Basak et al., 2021).

In this study, we characterized the closest homolog of MEB1 and MEB2 in Arabidopsis, namely MEB3 (AT4G27870). The function of MEB3 is thought to be unrelated to ER bodies because we previously showed that *MEB3* gene expression is not controlled by NAI1, a transcription factor regulating most ER body-related genes in seedlings (Yamada et al., 2013). Here, we investigated the subcellular localization and iron transport activity of MEB3 in Arabidopsis. Our results showed that MEB3 serves as a vacuolar, but not ER body-specific, iron transporter. The *MEB3* gene was expressed mainly in roots, and iron level were reduced in Arabidopsis *meb3* knockout mutants under iron-deficient conditions. Our findings suggest that MEB3 plays a role in iron accumulation in Arabidopsis root cells and is involved in root-to-shoot iron translocation in response to iron availability.

## 2 Materials and methods

### 2.1 Plant materials, plant growth condition, and yeast strains

*Arabidopsis thaliana* ecotype Columbia (Col-0) was used as the WT in this study. Arabidopsis T-DNA insertion mutants *meb3-1* (SALK_152844) and *meb3-2* (SALK_010196) were obtained from the Arabidopsis Biological Resource Center (ABRC; OH, USA). Seeds were surface-sterilized with 70% (v/v) ethanol and germinated photoautotrophically at 22°C under continuous light (approximately 100 µE s^-1^ m^-2^) on 1/2 MS medium (half-strength MS basal salt mixture [2623020; MP Biomedicals], 1% [w/v] sucrose, and 0.5% [w/v] MES-KOH [pH5.7]) containing 0.4% (w/v) Gellan Gum (Wako). For iron-deficit experiments, after 5 days, the seedlings were transferred to iron-sufficient medium (1/2 MS medium supplemented with100 µM NaFe(III)-EDTA) or no-iron medium (1/2 MS medium supplemented 100 µM Na_2_-EDTA instead of NaFe(III)-EDTA). Then, eight seedlings from each treatment were placed in separate square plates and incubated in a growth chamber for 16 days at 22°C under continuous light. Primary root length was measured using the ImageJ software. *Nicotiana tabacum* Bright Yellow 2 (BY-2) cell suspension cultures were grown in MS basal salt mixture (M5524; Sigma) supplemented with 3% (w/v) sucrose, 0.2% (w/v) KH_2_PO_4,_ 0.2 mg/L 2,4-dichlorophenoxyacetic acid, 1 mg/L thiamine, and 100 mg/L myo-inositol (pH 5.5) in the dark at 25°C on an orbital shaker (180 rpm). The *CCC1* and *ZRC1*-deficient yeast strain (Δ*ccc1*::KanMX4 and Δ*zrc1*::KanMX4; BY4741 background) was used for the heterologous expression of Arabidopsis *MEB1*, *MEB2*, *MEB3*, and *VIT1* cDNAs, to examine the subcellular localization patterns and iron transport activities of the encoded proteins.

### 2.2 Plasmid construction and plant transformation

To examine the subcellular localization of MEB3, a cDNA fragment of *MEB3* with stop codon was amplified and cloned into the Gateway Entry vector pENTR/SD/D-TOPO (Thermo Fisher Scientific) (**Supplementary Table S3**). The inserted gene was transferred into the destination vectors pGWB406 and pB5tdGW (gifts from T. Nakagawa), harboring *GFP* and *tdTOM* reporter genes, respectively (Nakagawa et al., 2007), using Gateway LR Clonase Enzyme mix (Thermo Fisher). The resultant constructs pB5tdGW/tdTOM-MEB3 and pGWB406/GFP-MEB3, carried *Prom35S*:*GFP-MEB3* and *Prom35S*:*tdTOM-MEB3*, respectively. The pB5tdGW/tdTOM-MEB3 construct was transformed into Col-0 plants via *Agrobacterium*-mediated transformation using the floral dip method (Clough and Bent, 1998). To perform functional analysis in yeast cells, full-length *VIT1* cDNA was amplified from the Arabidopsis cDNA library and subcloned into pENTR1A (Thermo Fisher) between the *Sal*I and *Eco*RV restriction sites. The DNA fragments containing *MEB3* (pENTR-MEB3) and *VIT1* (pENTR-VIT1) were cloned into the p415 GAL1-GW vector (Yamada et al., 2013) via the LR reaction to generate p415/MEB3 and p415/VIT1 vectors, respectively. To examine the subcellular localization of MEB1–3 proteins in yeast cells, full-length *MEB1*, *MEB2*, and *MEB3* cDNA were cloned into pAG416GAL-EGFP-ccdB (Addgene, plasmid # 14315) vector via the LR reaction. For GUS staining assay, the *MEB3* promoter region (2 kbp upstream from translational start codon) was amplified with specific primers (**Supplementary Table S3**) and cloned into pBI121 vector (Clontech) at *Hin*dIII and *Bam*HI sites.

### 2.3 Transient expression assays using Arabidopsis protoplasts

Protoplasts were isolated from 20 rosette leaves of 3-week-old plants using the tape-sandwich method described by Wu et al. (2009). The peeled leaves were gently shaken with enzyme solution for 90 min at room temperature. pGWB406/GFP-MEB3 was transiently expressed in protoplasts by polyethylene glycol (PEG)-mediated transfection (Yoo et al., 2007). The transfected protoplasts were incubated in the plant growth room (22°C, 16 h light/8 h dark cycle) for 1 day, harvested by centrifugation at 100 × *g*, and subsequently observed under a microscope. To isolate protoplasts from BY-2 cells, 1.5 g of 4-day-old cells was incubated in 10 mL enzyme solution (1% [w/v] cellulase Onozuka RS, 0.1% [w/v] Pectolyase Y-23, 0.4 M mannitol, 10 mM CaCl_2_, 5 mM MES-Tris [pH 5.5]) at 28°C for 1 h with gentle shaking.

### 2.4 Confocal microscopy

A confocal laser scanning microscope (LSM880; Carl Zeiss) was used to observe fluorescent proteins. GFP was detected using an argon laser (488 nm) and a 505/530 nm band-pass filter, chlorophyll *b* was observed using a helium-neon laser (633 nm) and 640/720 nm band-pass filter, and tdTOM signal was observed using a 561 nm diode-pumped solid-state laser and 560/640 nm band-pass filter. We used FM4-64 dye for either tonoplast or plasma membrane marker. The dye stains the plasma membrane or tonoplast depending on the difference in the incubation time (Yamada et al., 2005). To visualize the plasma membrane in plants, whole plants and protoplasts were incubated with 2 µM FM4-64 (Thermo Fisher) for 5 min. For tonoplast staining, 7-day-old seedlings were incubated in 4 µM FM4-64 dye for 30 min and then incubated in 1/2 MS medium for 3 hours. Yeast vacuolar membrane was stained with FM4-64 according to the method described in Desfougères et al., 2016. For DAPI staining, exponentially growing yeast cells were incubated with 2.5 µM DAPI (Thermo Fisher) for 1 hour at 30°C in the dark. Cells were washed with PBS buffer before observation. FM4-64 signal was observed using an argon laser (488 nm) and a 670/760 nm band-pass filter, and DAPI signal was observed using a blue-diode laser (405 nm) and 410/490 nm band-pass filter.

### 2.5 Fe probe and image quantification

Imaging of labile Fe^3+^ in Arabidopsis roots was performed according to Alcon et al. (2024) with minor modification. Seven-day-old seedlings were incubated in 10 mM MES-KOH (pH 5.7) with 10 µM 7-(4-methylpiperazin-1-yl)-4-nitrobenzo-2-oxa-1,3-diazole (MPNBD, a kind gift from T.C. Xiong) for 3 hours in the dark at room temperature. The fluorescence signal of activated MPNBD was observed using an argon laser (488 nm) and a band-pass filter (495-570 nm). The mature parts of root cells were observed and the average intensity of the insides of root cells was calculated by ImageJ software.

### 2.6 GUS activity staining

The 14-day-old plants harvested and placed in 90% (v/v) acetone for 15 min. Samples were washed three times with GUS staining buffer (50 mM sodium phosphate, 0.5 mM potassium ferrocyanide, 0.5 mM potassium ferricyanide, pH 7.2). The staining solution (1 mM 5-bromo-4-chloro-3-indolyl-*β*-D-glucuronide in GUS buffer) was added to the samples, and vacuum infiltrated for 3 min. After incubation at 37°C overnight, samples were rinsed in 70% (v/v) ethanol several times until plant pigments were removed.

### 2.7 Iron quantification

The total iron content of Arabidopsis seedlings, leaves and roots was quantified either using the iron colorimetric assay (Gao et al., 2021) or by inductively coupled plasma-mass spectrometry (ICP-MS) (Almario et al., 2017). To measure iron content by the colorimetric assay, first, the seedlings and roots were washed with deionized water to remove the remaining agar medium, and subsequently, the water was removed with a paper towel. Then, leaf and root samples were dried at 80°C for 20 h, and their dry weights were measured. To measure iron content by ICP-MS, 7-day-old seedlings and the leaves and roots of 21-day-old plants grown on 1/2 MS medium (50 µM Fe) were freeze-dried at -20°C overnight. The dried plant material was weighed and homogenized to a fine powder. Approximately 5 mg of the powdered plant material was digested with 500 μL of 67% (w/w) HNO_3_ in 15 mL Falcon tubes overnight at room temperature. Then, loosely closed tubes containing the samples were placed in a 95°C water bath until the liquid was completely clear (approximately 30 min). After being cooled to room temperature for 10–15 min, the samples were placed on ice, and 4.5 mL of deionized water was carefully added to the tubes. The samples were then centrifuged at 2,000 × *g* for 30 min at 4°C, and the supernatants were transferred into new tubes. The elemental concentration of iron was determined using Agilent 7700 ICP-MS (Agilent Technologies).

### 2.8 Functional analysis of MEB3 in yeast

The iron resistance assay was performed in yeast cells as described previously (Yamada et al., 2013). Briefly, 5 µL of yeast cell suspension (OD_600_ = 0.1 or 0.01) was spotted on SGal-agar medium containing 3 mM ammonium Fe(II) sulfate. For the manganese and zinc resistance assay, yeast cell suspension was spotted on SGal-agar medium containing different concentrations of manganese(II) chloride and zinc (II) sulfate. For liquid culture, yeast cell suspension was transferred to 3 mL of SGal liquid medium (OD_600_ = 0.05) containing 0 and 3 mM ammonium Fe(II) sulfate. The yeast cells were grown at 200 rpm and 30°C and monitored at various time intervals.

To measure total iron contents in yeast cells, yeast strains were grown overnight in YPD medium and then washed once with water. The washed cells were suspended in water and applied to 100 mL of SGal medium containing 0.5 mM ammonium Fe(II) sulfate. Cells were grown for 16 h at 30°C and then centrifuged at 5,000 rpm for 5 min. The precipitated cells were washed twice with 1 mM EDTA and once with water, and suspended in Milli-Q water. Subsequently, 1 mL of cells (OD_600_ = 10) was transferred into 1.5 mL tubes. After centrifugation at 10,000 rpm for 5 min, the cells are dried completely at 60°C for 3 days. The total iron content of yeast cells was measured by the iron colorimetric assay.

### 2.9 JA treatment

The leaves and roots of 2-week-old WT plants were cut and placed on the surface of water containing 50 μM methyl jasmonate (MeJA; Sigma-Aldrich). The JA treatment was performed for approximately 24 h at 22°C under continuous light.

### 2.10 RNA isolation and quantitative real-time PCR

To analyze mRNA expression levels in each plant organ, total RNA was isolated from 5-day-old cotyledons and the rosette leaves and roots of 14-day-old plants. After 2 weeks of germination, plants were transferred to soil and grown for 4 weeks. Then, total RNA was isolated from the cauline leaves, stems, green siliques, and flowers of 6-week-old plants. Isolation of total RNA from leaves and roots was performed using the TRIzol Reagent (Molecular Research Center, Cincinnati, USA), and isolation of total RNA from stems, green siliques, and flowers was performed according to the method described by Oñate-Sánchez and Vicente-Carbajosa (2009). The isolated total RNA was resuspended in distilled water and treated with DNase I (Thermo Fisher). Then, first-strand cDNA was synthesized from 2 µg of total RNA using Ready-to-Go RT-PCR beads (GE healthcare) and an oligo(dT) primer. The expression of *MEB1*, *MEB2*, *MEB3*, and *VIT1* was analyzed by qRT-PCR (QuantStudio 6, Thermo Fisher) using TaqMan Real-Time PCR Assays (Thermo Fisher), according to the manufacturer’s instructions. The transcript levels of *MEB1*, *MEB2*, *MEB3*, *VIT1*, *IRT1*, *FRO2*, *FIT*, *bHLH38*, *bHLH100*, *AHA2*, *NRAMP3*, *NRAMP4*, *FPN2*, *COPT2* and *FRO4* genes under iron-deficit conditions were analyzed using PowerUp SYBR Green Master Mix (Thermo Fisher). The gene-specific primer sets were designed with Primer3Plus software, and *UBQ10* was chosen as the housekeeping gene. The variation of total mRNA levels between the samples was normalized with constitutive expressing *UBQ10*. The expression of target genes was calculated using the amplification efficiency calibrated calculation method.

### 2.11 Immunoblot analysis

Total protein was extracted from 14 or 21-day-old roots by using 2 × sample buffer [20 mM Tris-HCl (pH 6.8), 40% (v/v) glycerol, 2% (w/v) SDS and 2% (v/v) 2-mercaptoethanol]. The 150 µL of sample buffer was added per 100 mg fresh weight root sample. The samples were heated at 94°C for 5 min. The 10 µL of the extract was subjected to SDS-PAGE (12% acrylamide gel). The immunoblot analysis was performed using anti-IRT1 (AS11 1780, Agrisera) antibodies.

## 3 Results

### 3.1 The VIT protein family consists of three subfamilies, and MEB3 belongs to the MEB subfamily

**Figure 1:**
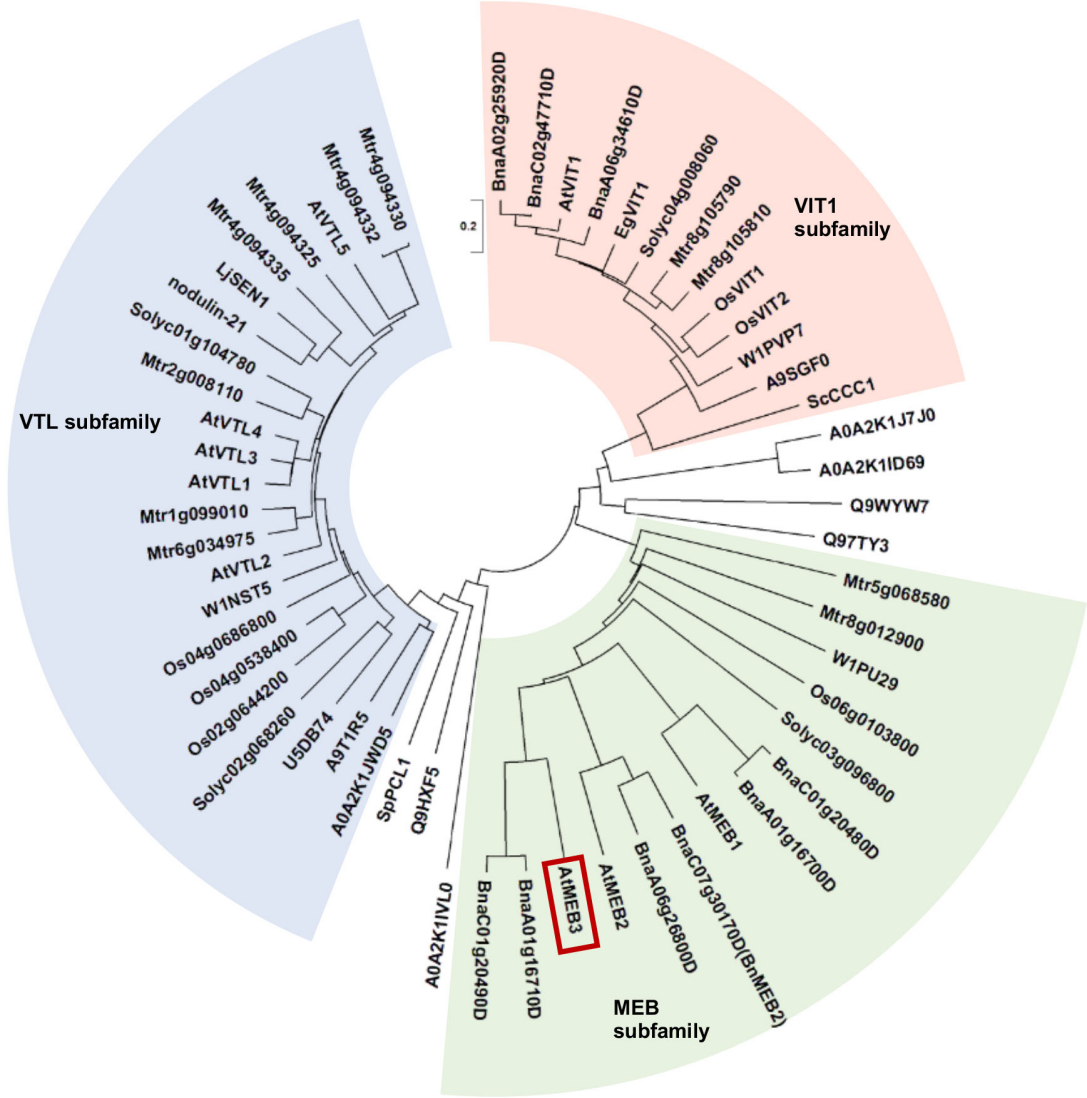
Phylogenetic tree of VIT family proteins. The three subfamilies, VIT1 (red background), VTL (blue background), and MEB (green background), are shown. Amino acid sequences of VIT1, MEB, and VTL subfamily proteins were obtained from the Protein BLAST and UniProt server. The sequences were aligned using the MUSCLE program with default settings in MEGA-X software. The phylogenetic tree was constructed using the neighbor-joining method. AtMEB3 is highlighted with a red box. The proteins used for the phylogenetic analysis are listed in **Supplementary Table S1**. The scale bar represents the evolutionary distance, expressed as the number of substitutions per amino acid.

Our previous analysis indicated that Arabidopsis possesses three highly homologous proteins, namely, MEB1, MEB2, and MEB3/At4g27870 (Yamada et al., 2013). These proteins have a multispanning transmembrane region that shows homology to VIT family proteins and is proposed to have a metal transporter function. This transmembrane region of VIT family proteins is dubbed DUF125. To estimate the functional similarity of VIT family proteins, we generated an unrooted phylogenetic tree of amino acid sequences harboring DUF125 (**Figure 1**; **Supplementary Table S1**). We selected several well-characterized plant species including a monocot (rice, *Oryza sativa*) and dicots (*M. truncatula* and *Brassica napus*), in which VIT family proteins have already been examined (Zhang et al., 2012; Walton et al., 2020; Zhu et al., 2016). In addition, we included representative terrestrial plant species, yeasts, and bacteria whose genomes have been sequenced and constructed a phylogenetic tree. The results showed that VIT family proteins could be divided into three subfamilies: VIT1, VTL, and MEB. The MEB subfamily contained proteins belonging not only to the Brassicaceae family (e.g., Arabidopsis and *B. napus*) but also to other plant families (**Figure 1**). Arabidopsis MEB1, MEB2, and MEB3 clustered in the MEB subfamily.

The EgVIT1 protein of eucalyptus (*Eucalyptus grandis*) contains four amino acid residues in DUF125, namely, Asp43, Glu72, Met80, and Tyr175, which together form a cavity for transporting iron (Kato et al., 2019). Additionally, amino acid substitution of Gly76 severely affected the iron transporter function of Arabidopsis VIT1 (Mary et al., 2015). Therefore, we investigated the presence of these amino acid residues in VIT family proteins (**Supplementary Figure S1**). We found that Asp43, Glu72, Met80, Tyr175, and Gly76 residues were well conserved in the VIT1 subfamily, while Glu72 and Tyr175 were not conserved in the VTL subfamily, and Asp43 and Glu72 were not conserved in the MEB subfamily. Collectively, these results suggest that the metal transport mechanisms of each subfamily may be slightly different between the VIT family proteins.

### 3.2 MEB3 functions as an iron and zinc transporter in yeast

**Figure 2:**
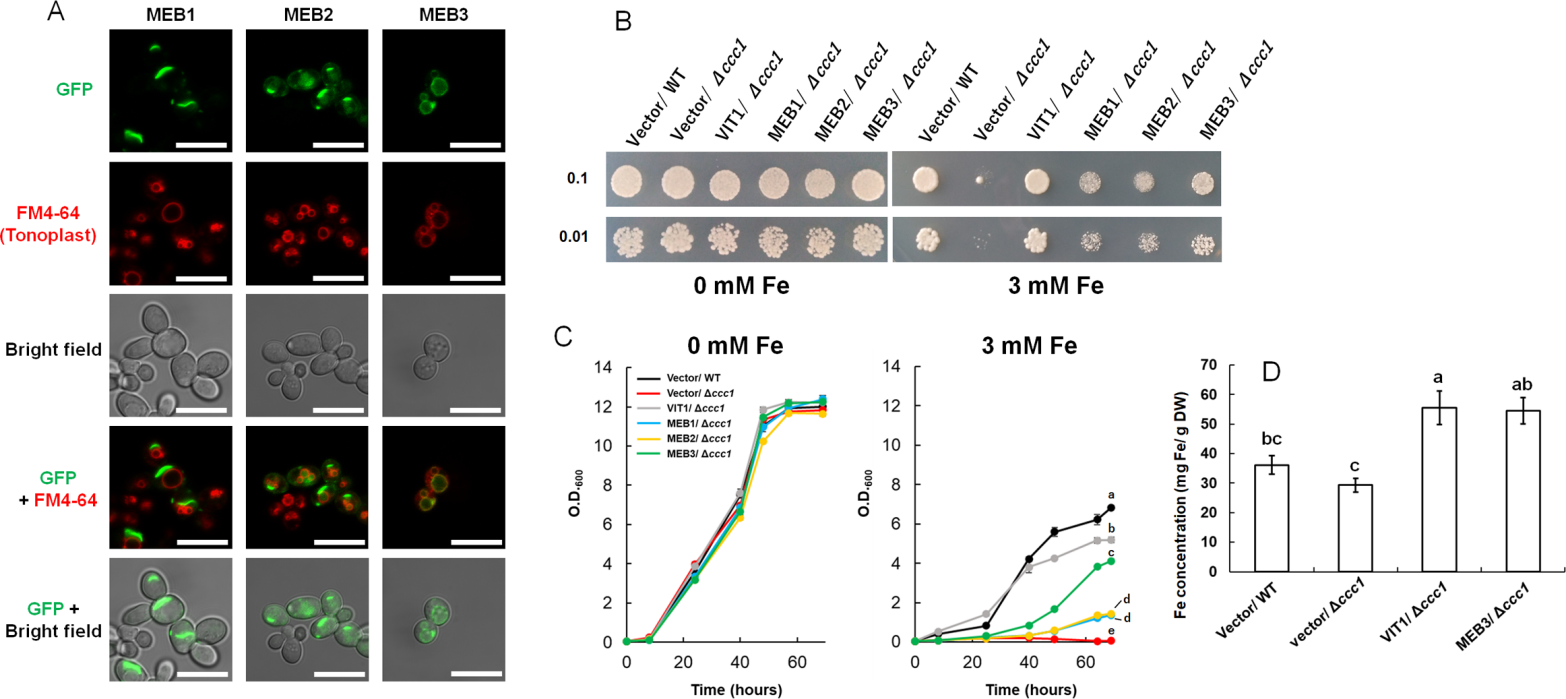
Protein subcellular localization, functional complementation, and iron accumulation were analyzed using yeast cells. **(A)** Subcellular localization assays of GFP-MEB1, GFP-MEB2, and GFP-MEB3 fusion proteins. Panels show confocal microscopy images and bright field images. The yeast tonoplast was stained with the vital dye FM4-64. Scale bars = 10 µm. **(B, C)** Functional complementation assay. The wild-type (WT) and iron-sensitive Δ*ccc1* strains of yeast were transformed with the empty vector (Vector) or vectors harboring *VIT1*, *MEB1*, *MEB2*, or *MEB3* and grown on SGal agar medium supplemented with or without 3 mM iron for 4 days **(B)**. Numbers on the left denote the concentration (OD_600_) of the spotted yeast cells. **(C)** six yeast strains were transferred to SGal liquid medium at OD_600_ 0.05 and grown with or without 3 mM iron. Different lowercase letters indicate significant differences determined using one-way ANOVA with a Tukey’s HSD test (*p* < 0.01; Tukey’s test). **(D)** Iron accumulation in yeast cells. The chart shows the total iron contents of yeast cells, as determined by the iron colorimetric assay, after 1 day of incubation on medium containing 0.5 mM (NH_4_)_2_Fe(SO_4_)_2_. Data represent mean ± standard error with biological replicates (SE; *n* = 3). Different lowercase letters indicate significant differences determined using one-way ANOVA with a Tukey’s HSD test (*p* < 0.05; Tukey’s test).

To examine whether MEB1–3 proteins exhibit iron transporter activity, we employed a yeast expression system. First, we checked the subcellular localization of MEB proteins in yeast by expressing the *MEB* genes as fusions with the *green fluorescent protein* (*GFP*) gene. The fluorescence of the GFP-MEB3 fusion protein overlapped with that of the tonoplast marker dye, FM4-64, but the vacuolar localizations of GFP-MEB1 and GFP-MEB2 fusion proteins were unclear; GFP-MEB1 appeared to form aggregates and GFP-MEB2 localized to the perinuclear ER membrane in yeast cells (**Figure 2A**). The aggregate structures in yeast cells overexpressing MEB1 and MEB2 did not co-localize with cell nuclei stained with 4’,6-diamidino-2-phenylindole (DAPI). (**Supplementary Figure S2**). Next, we used the vacuolar iron transporter deficient yeast mutant *ccc1* and tested whether the *MEB1*–*3* genes could recover the iron-sensitive phenotype of the mutant (**Figure 2B**). The yeast *ccc1* mutant showed normal growth on synthetic galactose (SGal) medium but reduced growth on SGal medium containing 3 mM iron, indicating that the *ccc1* mutant was unable to sequester the cytosolic iron in the vacuole, resulting in a cytosolic toxic iron level. Overexpression of *MEB1* and *MEB2* slightly improved the growth of the *ccc1* mutant on iron-containing medium (**Figure 2B**) (Yamada et al., 2013). The growth of the *ccc1* mutant was restored to a significantly higher level by *MEB3* overexpression than by *MEB1* or *MEB2* overexpression (**Figure 2B**). We also examined yeast growth in the liquid SGal medium containing 0 and 3 mM iron, and confirmed that *MEB3* overexpression significantly recovered yeast cell growth of *ccc1* mutant (**Figure 2C**). Considering the vacuolar localization of MEB3 in yeasts (**Figure 2A**), these results suggest that MEB3 transports iron into the vacuole to prevent cytosolic iron toxicity in the *ccc1* mutant. Finally, we measured the iron contents of yeast cells cultured in liquid medium containing 0.5 mM iron that allows *ccc1* mutant growth (**Figure 2D**). We found that overexpression of both *MEB3* and *VIT1* led to iron accumulation in *ccc1* mutant yeast cells, indicating that MEB3 enhances iron transport in the heterologous yeast expression system. CCC1 also works as a manganese transporter (Li et al., 2001), while ZRC1 is a vacuolar zinc transporter in yeast (Macdiarmid et al., 2002). We examined whether MEB3 and VIT1 overexpression could complement mutants deficient in these vacuolar manganese or zinc transporters in yeast (**Supplementary Figure S3**). The zinc resistance of the *zrc1* mutant was partly recovered by *MEB3* overexpression, suggesting that MEB3 has zinc transport activity. Manganese resistance was not recovered by *MEB3* overexpression in the *ccc1* mutant (**Supplementary Figure S3**). Conversely, the zinc resistance in the *zrc1* mutant was not recovered by *VIT1* overexpression, whereas the manganese resistance of the *ccc1* mutant was recovered by *VIT1* overexpression (**Supplementary Figure S3**).

### 3.3 MEB3 is a vacuolar membrane protein

**Figure 3:**
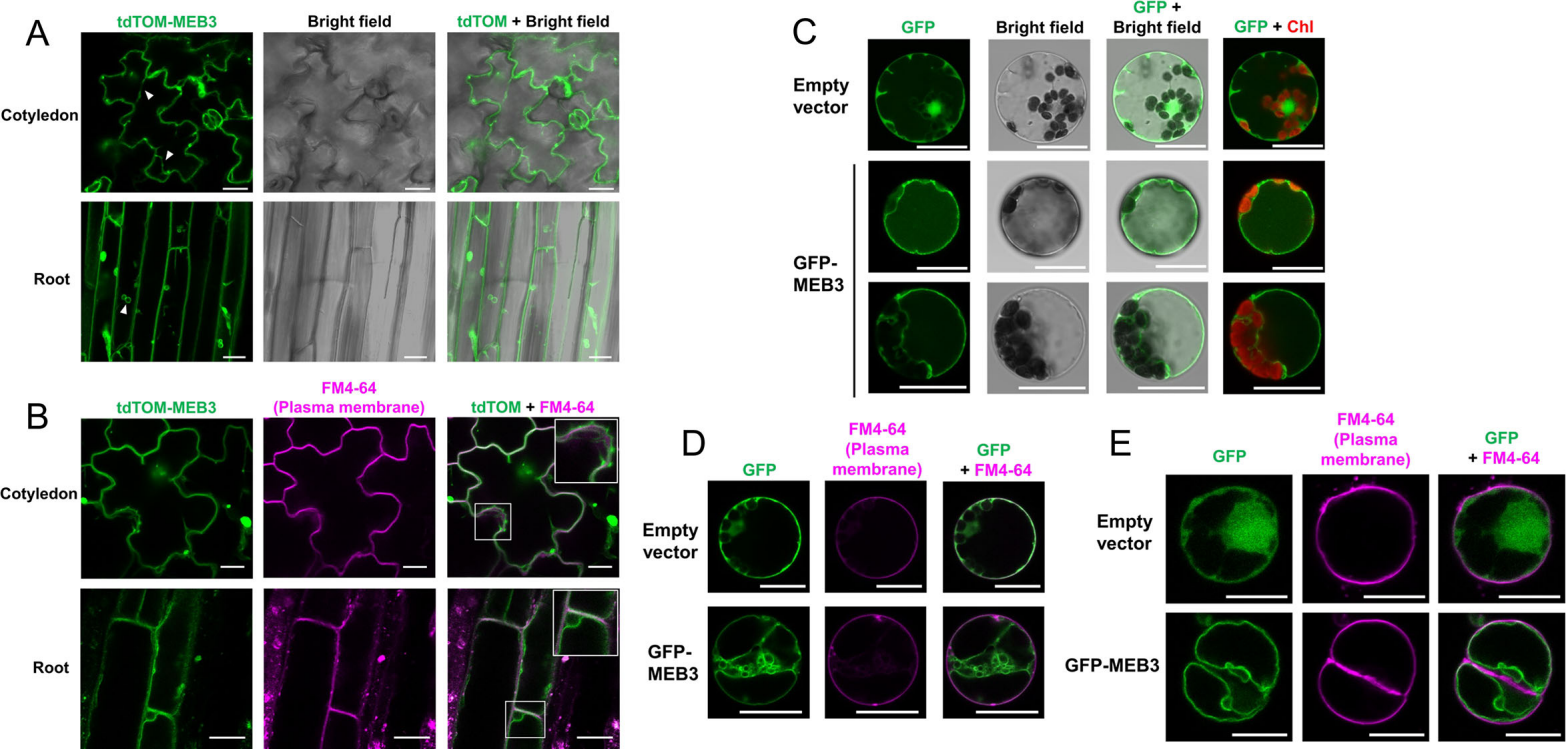
Determination of the tonoplast localization of MEB3 by confocal microscopy. **(A, B)** The image shows tdTOM fluorescence signals in the epidermal cells of cotyledons and roots of 5-day-old and 14-day-old transgenic plants, respectively. The fluorescent signal of tdTomato (Green) was pseudo-colored. Arrowheads show cytoplasmic strands **(A)**. Enlarged images are shown in the insets (squares in **B**). **(C-E)** Cytosolic localization of GFP (Empty vector) or GFP-MEB3 in transiently transformed Arabidopsis **(C, D)** and BY-2 **(E)** protoplasts. The plasma membrane was stained by short-time treatment with the vital dye FM4-64 **(B, D, E)**. ‘Chl’ indicates chlorophyll autofluorescence. Scale bars = 20 µm.

To investigate the subcellular localization of MEB3 *in planta*, we constitutively expressed a fusion protein comprising tandem dimer tomato (tdTOM), a fluorescent protein, and MEB3 (tdTOM-MEB3) under the control of the 35S promoter in Arabidopsis. tdTOM fluorescence was observed on membrane-bound structures of cotyledons and roots (**Figure 3A**). MEB1 and MEB2 localized to ER body membranes in Arabidopsis (Yamada et al., 2013). However, tdTOM fluorescence did not show ER body-like structures in cotyledons or roots where these structures should be abundant, indicating that there was no evidence of ER body localization of tdTOM-MEB3. This indicates that the subcellular localization pattern of MEB3 is different from that of MEB1 and MEB2. Notably, plants constitutively expressing *tdTOM-MEB3* showed fluorescence along transvacuolar strands and spherical structures in leaves and roots, respectively, suggesting that tdTOM-MEB3 mainly localizes to the tonoplast (**Figure 3A**); however, we could not exclude the possibility of plasma membrane-localization of MEB3 in this analysis. We visualized the plasma membrane with FM4-64 in cotyledon and root and found that tdTOM-MEB3 fluorescence did not overlap with FM4-64 fluorescence (**Figure 3B**). To investigate the subcellular localization of MEB3 in further detail, we transiently expressed the *GFP-MEB3* fusion in protoplasts isolated from Arabidopsis mesophyll cells (**Figures 3C, D**) and tobacco (*Nicotiana tabacum*) BY-2 culture cells (**Figure 3E**) after polyethylene glycol (PEG)-mediated transfection. In both cell types, the GFP-MEB3 fusion protein was detected on the tonoplast but not on the plasma membrane. These data indicate that Arabidopsis MEB3 is a tonoplast protein.

### 3.4 *MEB3* exhibits tissue-specific expression and its expression is mildly affected by jasmonic acid treatment

**Figure 4:**
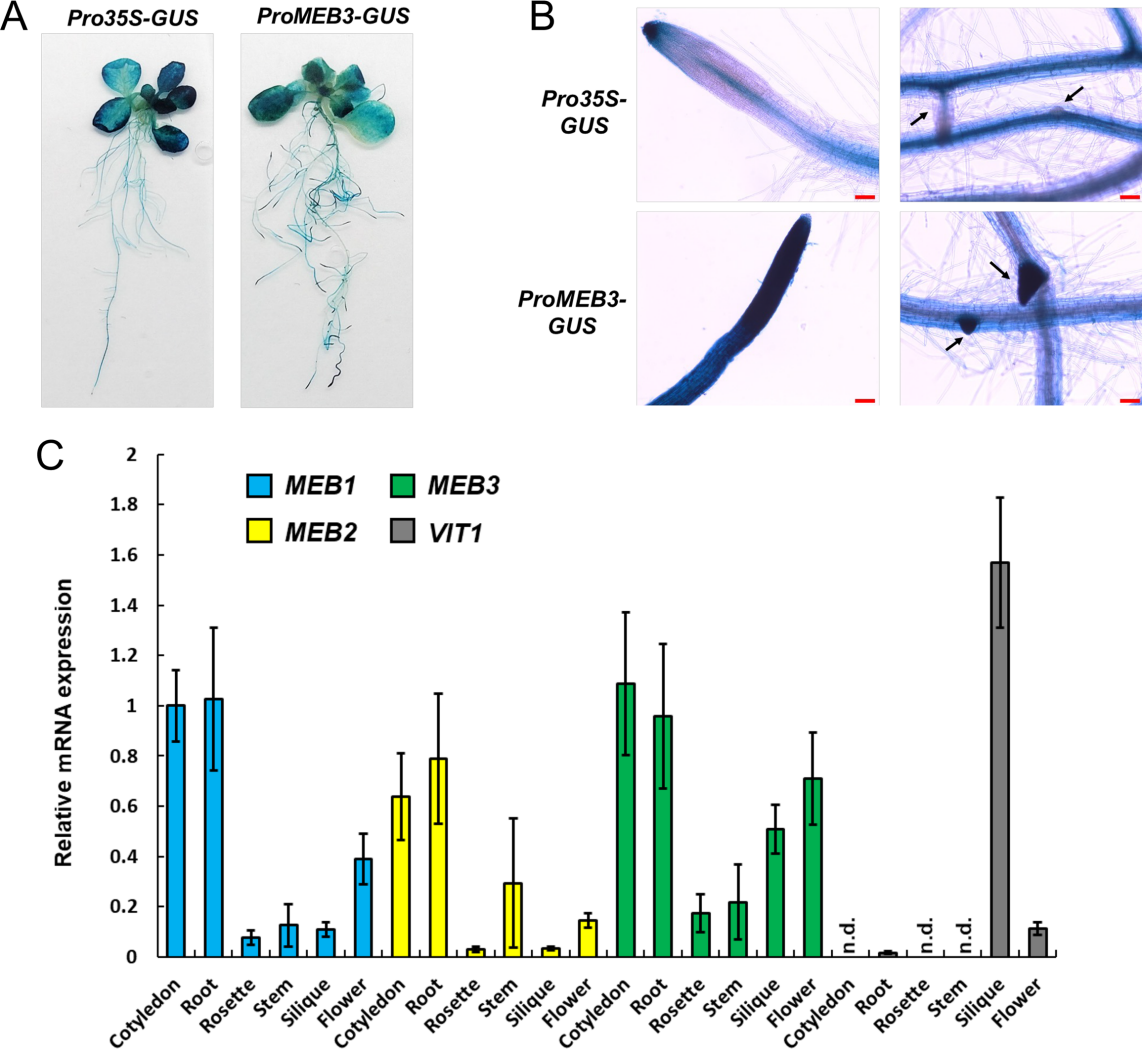
Expression analysis of *MEB1–3* and *VIT1* genes in Arabidopsis. **(A, B)** GUS histochemical staining of the 14-day-old transgenic lines expressing GUS gene driven by *35S* (*Pro35S-GUS*) and *MEB3* (*ProMEB3-GUS*) promoter. **(B)** The images show the meristematic zone of main roots (left) and the differentiation zone of lateral roots (right). The arrows indicate lateral roots. Scale bar = 100 µm. **(C)** Expression of *MEB1*, *MEB2*, *MEB3*, and *VIT1* genes in different organs of Arabidopsis plants. The chart shows relative mRNA expression (*MEB1* expression in the cotyledon was set as 1.0) in the cotyledons of 5-day-old seedlings; rosette leaves and roots of 14-day-old seedlings; and stems, siliques, cauline leaves, and flowers of 6-week-old plants. Data represent mean ± SE with biological replicates (*n* = 3). ‘n.d.’ indicates not detected.

To examine the organ-specific expression of *MEB3* in Arabidopsis plants, a transgenic plant harboring the *β*-glucuronidase (GUS) gene driven by the *MEB3* promoter was generated. *MEB3* promoter activity was detected in both leaves and roots (**Figure 4A**), and strong GUS activities were observed in the cell division and elongation zones of primary roots and lateral roots (**Figure 4B**). GUS activity staining was observed in the epidermal but not in the vascular tissues of roots.

We examined the expression patterns of *MEB1–3* and *VIT1* genes in different plant organs (**Figure 4C**). All *MEB* genes were highly expressed in the cotyledons and roots of 5- and 14-day-old plants, respectively. Expression pattern analysis of *MEB3* in organs showed that *MEB3* is expressed not only in roots and cotyledons but also in green siliques and flowers. The *VIT1* gene showed high expression level in green siliques; however, its expression could not be detected in other organs examined, as reported previously (Kim et al., 2006). These results suggest that MEB3 contributes to iron accumulation in cotyledons, roots, siliques, and flowers.

Treatment with JA, a plant defense hormone, increases the number of ER bodies in Arabidopsis (Ogasawara et al., 2009; Geem et al., 2019; Stefanik et al., 2020). Therefore, we hypothesized that the ER-body-related *MEB1* and *MEB2* expression would be increased by JA treatment, while non-ER-body-related *MEB3* expression would not respond to JA treatment, despite the homology between these genes. To test this hypothesis, we examined the expression of *MEB1*, *MEB2*, *MEB3* in Arabidopsis roots and leaves with and without JA treatment (**Supplementary Figure S4**). In rosette leaves, JA treatment induced the expression of all three *MEB* genes; however, the enhancement of *MEB3* expression was much lower than that of *MEB1* or *MEB2*. In roots, all three *MEB* genes were expressed before JA treatment; however, after JA treatment, the expression of *MEB1* and *MEB2* was slightly enhanced, whereas that of *MEB3* was not affected. Although MEB3 shares phylogeny with MEB1 and MEB2, the gene regulatory mechanism of *MEB3* differs from that of *MEB1* and *MEB2*, suggesting that MEB3 is not strongly related to ER bodies induced by the JA treatment. We examined *VIT1* expression under the same treatment conditions, but its expression was low in the leaves and roots irrespective of JA treatment (**Supplementary Figure S4**).

### 3.5 *MEB3* expression is upregulated by iron supplementation

**Figure 5:**
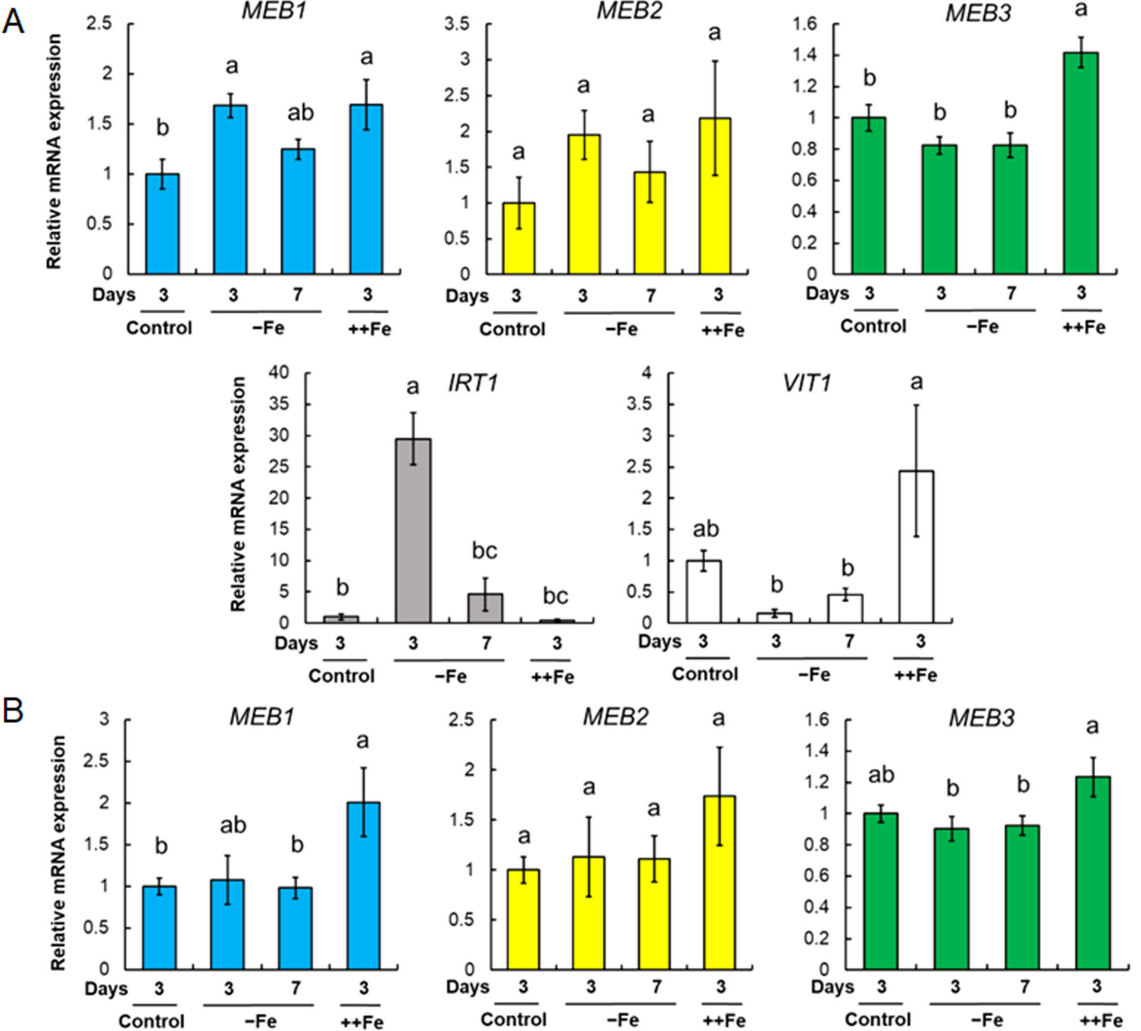
*MEB1*, *MEB2*, *MEB3*, *VIT1*, and *IRT1* gene expression under different iron concentrations. **(A, B)** Gene expression in roots **(A)** and shoots **(B)**. Plants were germinated in the normal medium (100 µM iron) for 7 days and then transferred to the normal (Control), no-iron (-Fe), or iron-sufficient (++Fe, 200 µM iron) medium for 3 or 7 days. The charts show the relative mRNA expression of each gene (expression in the 3-day control treatment was set as 1.0). The error bars indicate SE with biological replicates (*n* = 4). Different letters above the columns indicate significant differences determined using one-way ANOVA with a Tukey’s HSD test (*p* < 0.05).

The *IRT1* gene, which encodes a plasma membrane-localized iron transporter, is upregulated by iron depletion and downregulated by iron supplementation (Connolly et al., 2002; Vert et al., 2002). To investigate the response of other iron transporters under iron stress conditions, we examined changes in the expression levels of *MEB1*, *MEB2*, *MEB3*, *VIT1*, and *IRT1* in roots (**Figure 5A**) and shoots (**Figure 5B**) both in the presence and absence of iron. Seven-day-old wild-type (WT, Col-0) seedlings grown in iron-sufficient (100 µM) medium were transferred to no-iron (0 µM), iron-sufficient, or iron-excess (200 µM) medium for 3 and 7 days, and total RNA was isolated from each sample to examine gene expression. Consistent with previous reports (Connolly et al., 2002; Vert et al., 2002), *IRT1* expression in roots was strongly upregulated in no-iron medium and downregulated in iron-supplemented medium (**Figure 5A**). By contrast, the expression of both *MEB3* and *VIT1* genes in roots was downregulated in no-iron medium and upregulated in iron-excess medium. The expression levels of *MEB1* and *MEB2* in roots were upregulated under both no-iron and iron-excess conditions. The expression of all three *MEB* genes in shoots was slightly higher in plants grown in the iron-excess medium than in those grown in the control medium (**Figure 5B**). These results indicated that the regulation of vacuolar iron transporter gene (*MEB3* and *VIT1*) expression was different from that of the plasma membrane iron transporter (*IRT1*) and ER body iron transporter genes (*MEB1* and *MEB2*) in roots, suggesting that the mechanism regulating the expression of iron transporter genes is associated with the subcellular localization of the encoded proteins in roots.

### 3.6 *meb3* mutants exhibit reduced iron, cobalt, and copper accumulation in roots

**Figure 6:**
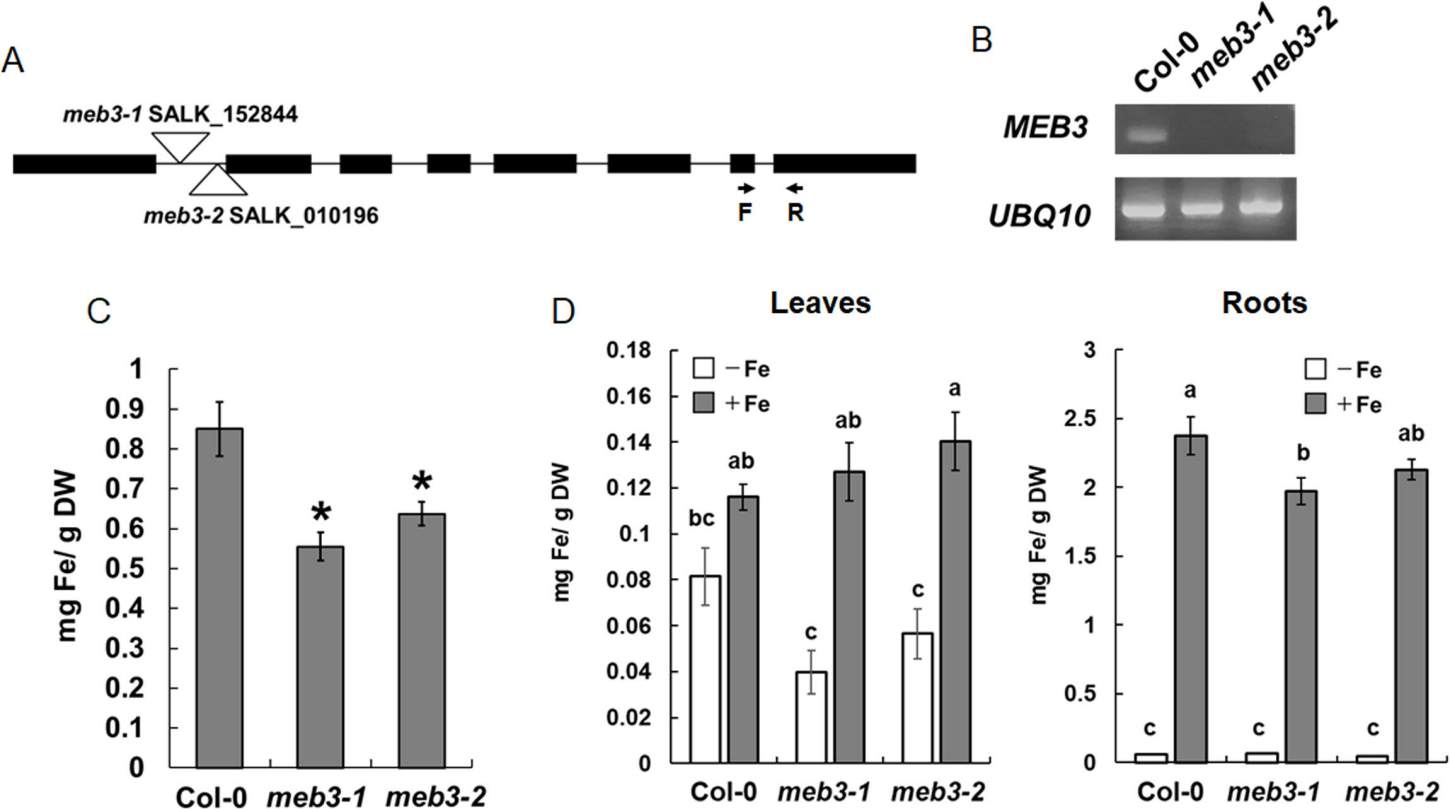
Loss of MEB3 affects the iron content in Arabidopsis leaves and roots. **(A)** T-DNA insertion site in *meb3-1* (SALK_152844) and *meb3-2* (SALK_010196). The arrows indicate the binding sites and orientations of forward (F) and reverse (R) primers, which were used to determine *MEB3* transcript levels in the mutants by RT-PCR. **(B)** Expression analysis of *MEB3* and *UBQ10* in the wild type (Col-0) and *meb3* mutants by RT-PCR. **(C)** Quantification of iron contents of 7-day-old wild-type, *meb3-1*, and *meb3-2* whole seedlings in 100 µM iron. **(D)** Changes of leaf (left) and root (right) iron contents in plants transferred to iron-sufficient or no-iron medium. Five-day-old seedlings were transferred from medium containing 50 µM iron to iron-sufficient (+Fe; 100 µM iron) and no-iron (-Fe; 0 µM) medium and grown for 16 days before the iron contents were measured. Iron levels were measured by iron colorimetric assay. Data represent mean ± SE with biological replicates (*n* = 4 in **(C)**, and 3 in **(D)**). Asterisk above the columns indicates significant differences between wild type and mutants based on Student’s t-test (p < 0.05). Different letters above the columns indicate significant differences based on one-way ANOVA with a Tukey’s HSD test (*p* < 0.05).

To examine the iron transport function of MEB3 in plants, we obtained two T-DNA insertion knockout mutants, *meb3-1* and *meb3-2*, in which the T-DNA was inserted in the first intron of *MEB3* (**Figure 6A**). Reverse transcription PCR (RT-PCR) analysis revealed no *MEB3* transcript in *meb3-1* and *meb3-2* mutants, indicating gene knockout (**Figure 6B**). Next, we measured the iron contents of whole seedlings grown on half-strength Murashige and Skoog (1/2 MS) medium containing 100 µM iron (**Figure 6C**) or iron contents of leaves and roots grown in the medium containing 50 µM iron (**Supplementary Figure S5**). Consistent with the higher expression of *MEB3* in cotyledons and mature plant roots (**Figure 4C**), iron accumulation was higher in WT seedlings (**Figure 6C**) and roots of 14-day-old WT plants than in their *meb3* mutant counterparts (**Supplementary Figure S5**), suggesting that MEB3 is important for iron accumulation in plants. Conversely, no statistically significant reduction in manganese and zinc levels was observed in the roots of *meb3* mutants compared with their WT counterparts (**Supplementary Figure S5**), but cobalt and copper levels were lower in *meb3-1* and *meb3-2* mutant roots than in WT roots. To address MEB3 function at the subcellular level, we examined vacuolar iron levels in the *meb3-1* and *meb3-2* mutant root cells. First, we checked if the *meb3* mutation affects vacuolar formation by staining the vacuolar membrane with FM4-64 (Yamada et al., 2005), and found no difference in vacuolar formation between WT and *meb3* mutant roots (**Supplementary Figure S6**). Next, we examined vacuolar iron accumulation using a chemical probe, 7-(4-methylpiperazin-1-yl)-4-nitrobenzo-2-oxa-1,3-diazole (MPNBD) (Alcon et al., 2024) that emits green fluorescence in the presence of Fe^3+^, which is thought to replete in the vacuole (Sági-Kazár et al., 2022). The vacuoles and symplasts of root cells were positive for MPNBD fluorescence as reported previously (Alcon et al., 2024), but MPNBD fluorescence was lower in the vacuoles of *meb3* mutants than in those of the WT (**Supplementary Figure S7**), indicating vacuolar iron accumulation was reduced in *meb3* mutants.

Plants absorb iron from the soil, store it in roots, and distribute it to shoots via long-distance transport. Root iron storage may balance leaf iron distribution in response to iron availability in the soil. To determine whether MEB3 is responsible for root iron storage under the iron-sufficient condition and the control of leaf iron distribution in response to later iron availability, we transferred 5-day-old WT and *meb3* mutant seedlings from the germination medium (50 µM iron) to iron-sufficient (100 µM) or no-iron (0 µM) growth medium, and measured root and leaf iron levels after 21 days. In WT plants, the root iron level was remarkably higher in plants transferred to iron-sufficient medium than in plants transferred to no-iron medium, whereas leaf iron level was only modestly higher on iron-sufficient medium than on no-iron medium (**Figure 6D**; **Supplementary Figure S8**). These results indicate that roots act as an iron storage organ, and root iron storage buffers leaf iron levels. We observed a similar trend in leaf iron level changes in *meb3* mutants, but surprisingly, the leaf iron level in *meb3* mutants was lower in plants transferred to no-iron medium and higher in plants transferred to iron-sufficient medium than in the WT. Consistent with previous observations (**Supplementary Figure S5**), the iron level in *meb3* roots was lower than that in WT roots in iron-sufficient medium (**Figure 6D**). These findings indicate that the iron storage capacity, and consequently buffering function, of roots was reduced in the *meb3* mutants. Together, these results suggest that MEB3 contributes to iron translocation across organs in Arabidopsis.

### 3.7 MEB3 mutation may affect root and shoot growth under iron-deficit conditions

**Figure 7:**
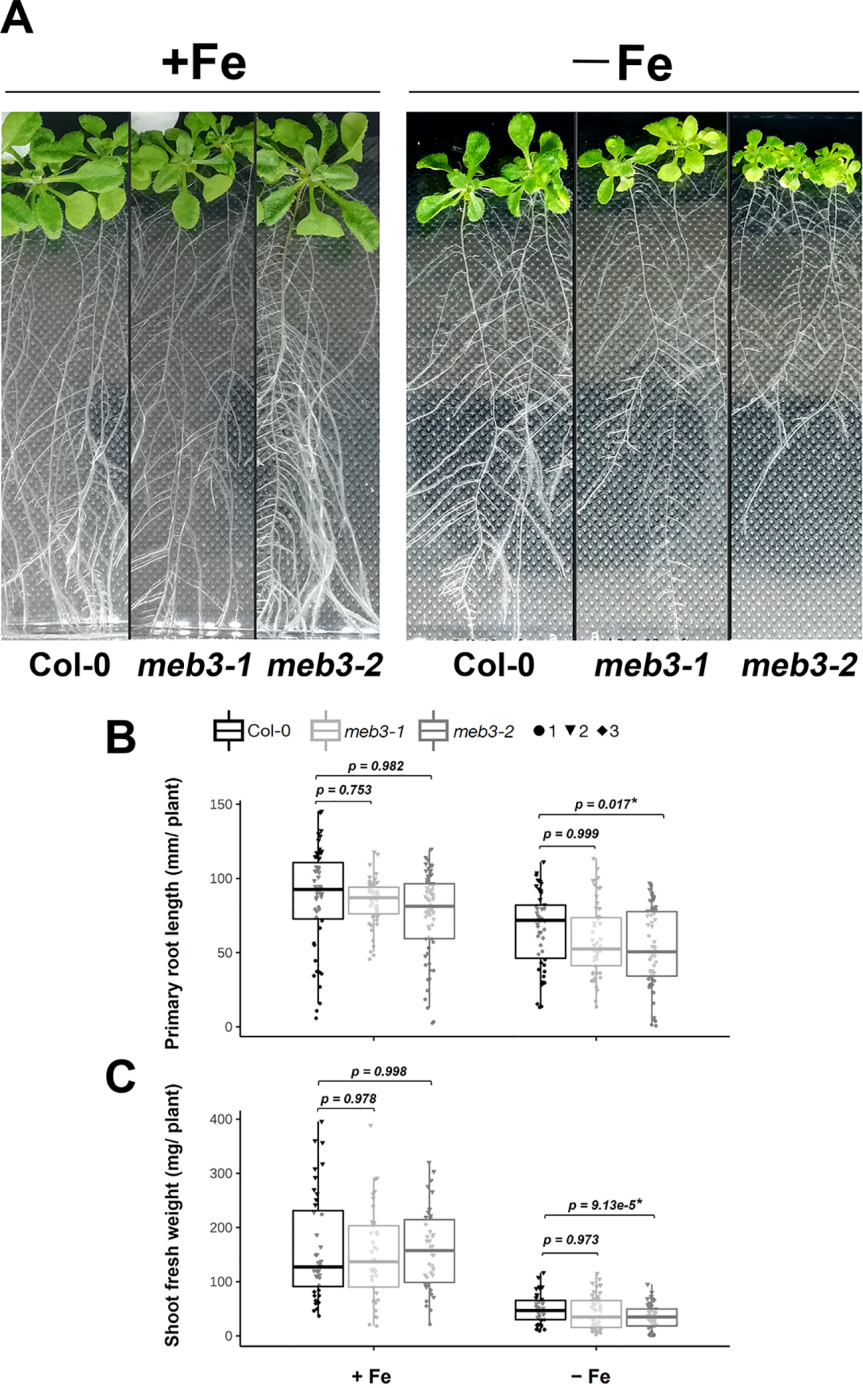
Growth phenotype of *meb3* knockout mutants. **(A)** Photograph of 21-day-old wild-type (Col-0), *meb3-1*, and *meb3-2* plants. Five-day-old seedlings grown in ½ MS including 50µM Fe-EDTA were transferred to 100 µM Fe-EDTA or Fe-free medium and grown for 16 days. **(B, C)** Primary root length **(B)** and shoot fresh weight **(C)** of Col-0, *meb3-1*, and *meb3-2* plants treated as the same as in **(A)**. Asterisks indicate significant differences between Col-0 and mutant plants. Circle, triangle, and diamond represent values of biological replicates. The *p*-values displayed on the boxplot were obtained using one-way ANOVA with a Tukey’s HSD test applied over log2-transformed data.

Because the iron storage capacity of *meb3* mutant roots was reduced, we hypothesized that the growth of *meb3* mutant plants would be affected when plants are transferred to iron-deficit conditions from iron-sufficient conditions. To test this hypothesis, we transferred 5-day-old WT and *meb3* mutant seedlings from the germination medium (50 µM iron) to iron-sufficient (100 µM) or no-iron (0 µM) growth medium, and measured primary root length and shoot fresh weight after cultivation for 21 days. The primary root lengths of WT, *meb3-1*, and *meb3-2* plants were similar in plants transferred to iron-sufficient medium, but the primary root length of the *meb3-2* mutant was slightly reduced compared with those of WT plants transferred to no-iron medium (**Figures 7A, B**). Similar trends were observed for shoot fresh weight (**Figures 7A, C**) on iron-sufficient and no-iron medium. However, the effect of plant growth in the *meb3-1* mutant was not significant. These findings suggest that the MEB3-mediated vacuolar iron storage may assist plant growth when plants are exposed to iron deficiency, but other iron storage, e.g. cell wall stored iron, contribute as iron sources to cover the impact of *meb3* mutation.

We found that *MEB3* expression was increased after 3 days of excess iron (200 µM) treatment (**Figure 5**). Therefore, we examined the plant growth of WT and *meb3* mutants in the iron-excess (200 µM) condition. The shoot growth of the WT and *meb3* mutants was reduced when 5-day-old plants were further germinated for 16 days in the 200 µM iron condition, confirming that this level of iron is stressful for Arabidopsis growth. However, the primary root lengths and shoot fresh weights tended to reduce but not significantly in *meb3* mutants relative to those in the WT (**Supplementary Figure S9**), implying that *MEB3* deficiency enhances iron toxicity in plants.

### 3.8 MEB3 is involved in the regulation of iron homeostasis genes in iron-sufficient conditions

**Figure 8:**
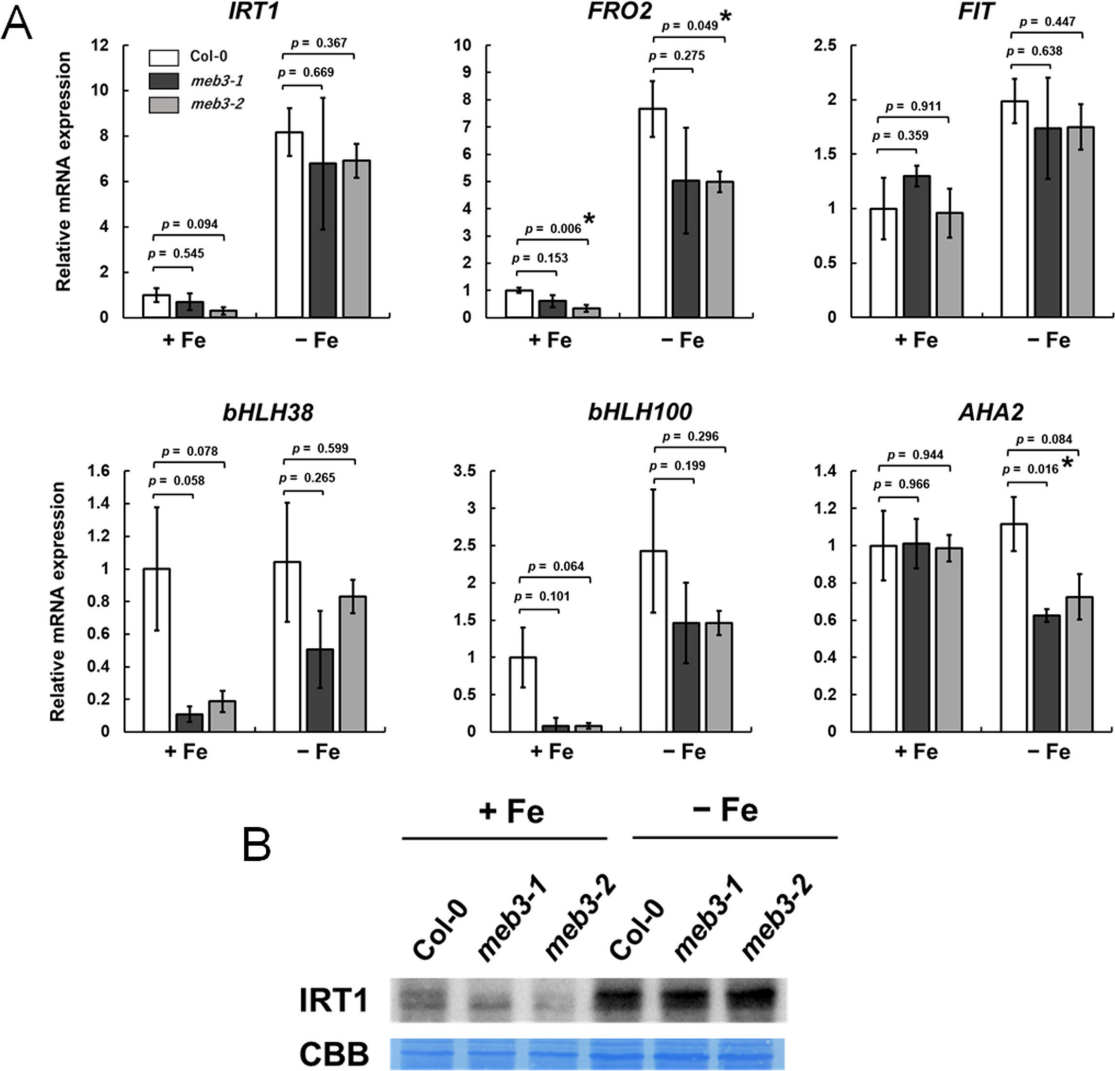
Expression of iron homeostasis genes and IRT1 protein levels in the roots of the *meb3* mutant. Plants were germinated in normal medium (50 µM iron) for 5 days and then transferred to normal (+Fe, 100 µM iron) or no-iron (-Fe) medium for 16 days. **(A)** the charts show the relative mRNA expression of each gene (expression in Col-0 +Fe was set as 1.0). The error bars indicate SE with biological replicates (*n* = 4). Asterisks above the columns indicate significant differences with respect to Col-0 under each iron condition based on Student’s *t*-test (p < 0.05). **(B)** Immunoblot analysis of wild-type and mutant roots was performed using the IRT1 antibody. Coomassie blue staining served as a loading control.

We found that the shoot and root iron levels in *meb3* mutant plants changed when they were transferred from normal growth conditions to iron-sufficient or iron-deficit conditions (**Figure 6D**) and grown for 16 days. Therefore, we examined whether the expression of iron uptake genes, namely *IRT1*, *FRO2*, and *AHA2*, and their transcription factor genes, namely *FIT*, *bHLH38*, and *bHLH100*, was affected in *meb3* mutant plants 16 days after their transfer to iron-sufficient or no-iron medium. In iron-sufficient medium, the mRNA expression of *bHLH38* and *bHLH100* was reduced, while *IRT1* and *FRO2* gene expression and IRT1 protein accumulation tended to be, and was slightly reduced, respectively, in *meb3* mutant roots relative to that in WT roots (**Figures 8A, B**). *FIT* and *AHA2* expression was not reduced in the *meb3* mutant roots relative to that in WT roots (**Figure 8A**). These results indicate that disruption of *meb3* affects IRT1 protein accumulation, as well as *bHLH38* and *bHLH100* gene expression, which may partially explain the reduction in root iron accumulation observed in the *meb3* mutants (**Figure 6D**).

When the plants were grown for 16 days under the no-iron medium, the expression of these iron homeostasis genes tended to be, but not significantly lower in *meb3* mutant roots (**Figure 8A**). The IRT1 protein levels in the *meb3* mutant were almost the same as those in WT plants under the iron-deficit condition. These results indicate that MEB3 deficiency may reduce but not largely change the expression of iron homeostasis and uptake genes. We examined the expression of vacuolar iron importer and exporter genes, *NRAMP3*, *NRAMP4*, *FPN2*, and *VIT1*, under the same experimental condition (**Supplementary Figure S10**). The *NRAMP3* and *NRAMP4* expression levels were not different between plants grown on a no-iron medium and iron-sufficient medium in our experimental condition, although it has been reported that *NRAMP3* and *NRAMP4* respond to iron deficiency. We speculate that the expression of *NRAMP3* and *NRAMP4* had declined to the basal levels after plants were grown in the no-iron medium for 16 days since the response of *NRAMP4* was not as strong as that of *IRT1* and *FRO2* under iron-deficient conditions (Stein and Waters, 2012). The expression of these genes was not markedly different between WT and *meb3* mutant roots (**Supplementary Figure S10**). bHLH38 and bHLH100 also regulate the expression of copper uptake genes, namely *COPT2* and *FRO4*, and expressions of these genes were significantly reduced in *meb3* mutant roots under iron-sufficient conditions (**Supplementary Figure S11**).

## 4 Discussion

### 4.1 Arabidopsis MEB3 is a vacuolar iron transporter

Arabidopsis MEB3, like MEB1 and MEB2, showed iron transport activity when expressed in yeast. However, we found that MEB3 localized to the tonoplast, but not to ER bodies, in Arabidopsis leaves and roots. Thus, our results suggest that unlike MEB1 and MEB2, which function as ER body-localized iron transporters (Yamada et al., 2013), Arabidopsis MEB3 functions as a vacuolar iron transporter.

In budding yeast, the vacuolar iron transporter CCC1 controls cytosolic iron level, and the *ccc1* mutant does not grow in iron-containing medium, because of cytosolic iron toxicity (Kim et al., 2006; Li et al., 2001). Overexpression of Arabidopsis *VIT1* and *MEB1–3* complemented the growth inhibition phenotype of the *ccc1* mutant (**Figures 2B, C**), indicating that VIT1 and MEB1–3 proteins act as iron transporters and remove iron from the cytosol. However, the growth of *MEB1*- and *MEB2*-expressing *ccc1* mutant cells was lower than that of *VIT1*- and *MEB3*-expressing yeast, suggesting that the iron transport function of MEB1 and MEB2 is less strong than that of VIT1 and MEB3. Additionally, we found that MEB1 and MEB2 localized to the ER, while VIT1 and MEB3 localized to the tonoplast in yeast cells (**Figure 2A**). Therefore, the functional difference between MEB1/MEB2 and VIT1/MEB3 could be attributed to the difference in their subcellular localization patterns rather than the difference in their transporter activities; the iron accumulation capacity of vacuoles is higher than that of the ER.

Amino acid residues crucial for the iron transport activity of EgVIT1 (Kato et al., 2019) were conserved in the VIT1 subfamily, but some of these residues were not conserved in the VTL and MEB subfamilies (**Supplementary Figure S1**). Nevertheless, Gollhofer et al. (2014) showed that two VTL subfamily proteins, namely, Arabidopsis VTL1 and VTL2, exhibit iron transport activity. Consistently, our data indicated that Arabidopsis MEB1, MEB2, and MEB3 are involved in iron transport (**Figure 2B**). The VTL subfamily proteins regulate iron transport into the symbiosome in the root nodules of soybean and alfalfa plants (Brear et al., 2020; Liu et al., 2020; Walton et al., 2020). These findings indicate that the conservation of these amino acid residues is not crucial for the iron transport activity of VTL and MEB subfamily proteins, which suggests that their iron transport mechanism is unique.

In addition to its iron transport activity, Arabidopsis VIT1 also has manganese transport activity (Kim et al., 2006). We confirmed the manganese transport activity of VIT1 but could not find such activity for MEB3 using yeast cells (**Supplementary Figure S3**). Instead, we found that MEB3, but not VIT1, has weak zinc transport activity. These results indicate that the metal transport mechanism and substrate specificity of MEB3 are different from those of VIT1. MEB3 may facilitate vacuolar storage of zinc in plants, but it is not the primary factor for zinc accumulation in plants, based on the results obtained using *meb3* mutant plants.

Our results suggest that Arabidopsis MEB3 is a vacuolar protein, implying the existence of a mechanism that distinguishes between the subcellular localization of MEB3 and MEB1/2 in Arabidopsis. Surprisingly, the *B. napus* ortholog of MEB2 localizes to the tonoplast but not to ER bodies (Zhu et al., 2016), indicating that the subcellular localization of MEB proteins is not conserved even within the Brassicaceae family. The vacuolar transport signal of MEB3 and BnMEB2 remains to be identified. Wang et al. (2014) showed that VIT1 possesses a dileucine motif (D/EXXXLL), which leads to the vacuolar targeting of VIT1. This motif also exists in NRAMP3 and NRAMP4 and is responsible for their vacuolar localization (Müdsam et al., 2018). However, we could not find the D/EXXXLL motif in MEB3. The MEB subfamily proteins harbor a long motif in the N-terminal region, which is more varied in sequence than the transmembrane domain in their C-terminal region (Yamada et al., 2013). This uncharacterized motif in the N-terminal region might determine the subcellular localization of MEB subfamily proteins in plants.

### 4.2 MEB3 contributes to iron accumulation in roots

The expression level of *MEB3* was higher in cotyledons and roots than in other tissues (**Figure 4C**), indicating that MEB3 functions in cotyledons and roots. Additionally, the root iron content of *meb3* mutants was lower than that of the WT (**Figure 6D**; **Supplementary Figure S5**), indicating that MEB3 is involved in iron accumulation in Arabidopsis roots. Over 50% of iron exists as apoplastic (Liu et al., 2023), but there are certain levels of vacuolar iron (Eroglu et al., 2016; Lanquar et al, 2010), which support the observation that *meb3* deficiency reduced total iron levels in roots. Furthermore, the mRNA expression of *bHLH38*, and *bHLH100* was reduced while IRT1 protein expression was slightly reduced in *meb3* mutants grown in iron-sufficient medium (**Figure 8**). The reduced accumulation of IRT1 may negatively affect iron uptake from iron-sufficient medium and the total iron level in the root. Therefore, we speculate that the reduced iron accumulation in *meb3* mutant roots in plants grown in iron-sufficient medium can be explained by reduced vacuolar iron storage levels and reduced iron uptake from the medium.

In Arabidopsis, iron accumulates in the epidermal cells of the primary root, and is abundantly detected in root apical and lateral root meristems (Reyt et al., 2015). Interestingly, we found that the *MEB3* promoter is activated in the root epidermis and strongly activated in the root tip of primary and lateral roots (**Figure 4B**), indicating that *MEB3* expression and iron accumulation patterns overlap in Arabidopsis roots. These findings suggest that MEB3 might contribute to iron accumulation and the distribution patterns of iron in the root.

Besides Arabidopsis MEB3, VTL subfamily proteins are also involved in root vacuolar iron accumulation (Rodríguez-Haas et al., 2013; Ram et al., 2021). Arabidopsis *vtl3* and *vtl5* mutants exhibit reduced root iron contents compared to the WT (Gollhofer et al., 2011). Arabidopsis FPN2 is a vacuolar iron transporter that does not belong to the VIT family but is expressed in roots (Morrissey et al., 2009). Therefore, one can expect that vacuolar iron transporters, FPN2, VTL1–5 (Chen et al., 2023) and MEB3 regulate vacuolar iron contents under iron-sufficient conditions in a functionally redundant manner, which may be why the *meb3* mutants do not show a complete loss of root iron and growth phenotype.

The flexibility of root iron accumulation helps plants to grow under iron stress conditions. Expression of the plasma membrane iron transporter gene *IRT1* was suppressed in Arabidopsis seedlings and roots in a medium containing excess iron (**Figure 5A**), suggesting that plants avoid taking up unwanted iron from the medium. By contrast, the expression of two vacuolar iron transporter genes, *OsVIT2* and *BnMEB2*, was upregulated in roots under excess iron conditions (Aung et al., 2018; Aung and Masuda, 2020; Zhu et al., 2016), suggesting that plants increase the abundance of vacuolar iron transporters to enhance the sequestration of cytosolic iron in vacuoles. In the current study, the expression of Arabidopsis *MEB3* and *VIT1* genes was upregulated by excess iron (**Figure 5A**), suggesting that MEB3 and VIT1 participate in vacuolar sequestration of cytosolic iron under excess iron conditions. The function of VIT1 in Arabidopsis roots is still obscure, but considering that the expression level of *VIT1* was lower than that of *MEB3* in the root (**Figure 4**; **Supplementary Figure S4**), the contribution of VIT1 to iron homeostasis may not be as strong as that of MEB3 in Arabidopsis roots.

The genes involved in iron uptake tended to have lower mRNA levels in *meb3* mutants than in the WT (**Figure 8**), suggesting that the *meb3* mutants may reduce iron uptake capacity from the medium by reducing the expression of these genes. Several transcription factors, including FIT and type Ib bHLHs (bHLH38, bHLH39, bHLH100, bHLH101), regulate the expression of iron uptake genes (Gao and Dubos, 2021). Indeed, *bHLH38* and *bHLH100* expression was reduced in *meb3* mutant roots (**Figure 8A**). Therefore, we speculate that the absence of vacuolar iron sequestration in the *meb3* mutant activates an excess-iron sensing system to suppress the expression of transcription factors regulating the expression of iron homeostasis genes in roots. Although the role of MEB3 in the iron sensing system is still unclear, our findings highlight the complexity of iron homeostasis mediated by the root iron storage function of MEB3.

In *meb3* mutants, cobalt and copper levels were decreased in roots (**Supplementary Figure S5**), suggesting that MEB3 may affect the accumulation of these metals in plants. However, we were unable to conclude that MEB3 has cobalt and copper transport activities because we could not exclude the possibility that MEB3 disruption changes iron homeostasis to reduce the expression of other metal transporter genes in *meb3* mutants. Indeed, bHLH38 and bHLH100 can induce the expression of copper uptake genes, *COPT2*, *FRO4*, and *FRO5* (Chia and Vatamaniuk, 2024), and the expression of *bHLH38, bHLH100, COPT2* and *FRO4* genes was reduced in *meb3* mutant roots (**Figure 8A**; **Supplementary Figure S11**). Moreover, we found that the accumulation of IRT1, which involved in cobalt accumulation in roots (Barberon et al., 2011; Vert et al., 2002) was reduced in *meb3* mutants.

Treatment with JA, a defense-related hormone known to induce ER body formation (Stefanik et al., 2020), modestly induced *MEB3* expression in leaves (**Supplementary Figure S4**). By contrast, the expression of *MEB1* and *MEB2*, which encode ER body-localized proteins (Yamada et al., 2013), was strongly induced by JA treatment. This suggests that the function of MEB3 *in planta* is different from that of MEB1 and MEB2; for example, MEB3 may not be involved in plant defense, unlike MEB1 and MEB2.

### 4.3 MEB3 has a potential role in the root-to-shoot translocation of iron

We showed that MEB3 is involved in root iron accumulation, and that the root iron level of *meb3* mutants was lower than that of the WT in normal medium (**Figure 6C**). Although *MEB3* expression was not high in shoots, the shoot iron levels decreased and plant growth reduced in *meb3* mutants compared with WT plants upon transfer from the normal medium to the no-iron medium (**Figure 6D**). Because iron was absent from the medium, the transferred plants used root-storage iron for growth in this experiment. Therefore, we speculate that the tendency of growth reduction in *meb3* mutants may be attributed to the reduced capacity for root iron storage and iron uptake in this mutant. These findings suggest that root iron accumulation capacity may affect shoot growth in the iron-deficient condition, presumably by the mobilization of root iron reserve to the shoot.

In Arabidopsis, it has been postulated that vacuolar NRAMP3 and NRAMP4 proteins are involved in unloading iron from the vacuoles into the cytosol (Lanquar et al., 2005), and a part of unloaded cytosolic iron chelates with NA (Klatte et al., 2009). The expression of genes involved in the iron remobilization pathway, such as *NRAMP3*, *NRAMP4*, and NA biosynthesis, is enhanced in response to iron deficiency (Lanquar et al., 2005; Klatte et al., 2009). By contrast, the expression of *MEB3* and *VIT1* tended to be reduced (**Figure 5**). This finding is consistent with the results of Gollhofer et al. (2011), who showed that the expression of *VTL1* and its homologs is reduced in roots in response to iron deficiency. Therefore, it is plausible that vacuolar iron influx is reduced, and vacuolar iron remobilization is increased, in roots for efficient iron translocation to the shoot under iron-deficient conditions.

In iron-sufficient medium, root iron accumulation was reduced, but shoot iron levels were slightly enhanced in the *meb3* mutant, indicating that the root-to-shoot transport of iron increases in *meb3* mutants under iron-sufficient conditions (**Figure 6D**). This finding suggests that MEB3-mediated iron storage capacity in the root is responsible for buffering leaf iron levels when the amount of iron in the environment changes dramatically. Therefore, MEB3-mediated vacuolar iron storage in Arabidopsis roots has two functions: 1) storage of iron to prepare for iron shortage in the shoot and 2) buffering shoot iron levels by storing iron in the root.

Proper iron distribution is essential for plant growth. For example, Arabidopsis *vit1* mutant shows no difference in iron contents relative to the WT but exhibits impaired iron accumulation in provascular strands in seeds (Kim et al., 2006). The unusual distribution of iron in the *vit1* mutant affects seedling development under iron-deficient conditions (Chu et al., 2017). Similarly, we found that MEB3 is involved in the proper distribution of iron between shoots and roots, and that its absence affects plant growth and the expression of genes involved in iron homeostasis. Considering that MEB subfamily proteins are present in almost all plant species in addition to the ER body-producing plants, we propose that these proteins are involved in similar functions (i.e., vacuolar iron accumulation) to promote plant growth.

## Data Availability

The raw data supporting the conclusions of this article will be made available by the authors, without undue reservation.
