# Supplementary material for: Arabidopsis MEB3 functions as a vacuolar metal transporter to regulate iron accumulation in roots
Source: Front Plant Sci. 2025 Mar 6;16:1517144. doi: 10.3389/fpls.2025.1517144 (PMC11922923; doi:10.3389/fpls.2025.1517144)
Supplement: Supplementary file 1 [file DataSheet1.pdf]

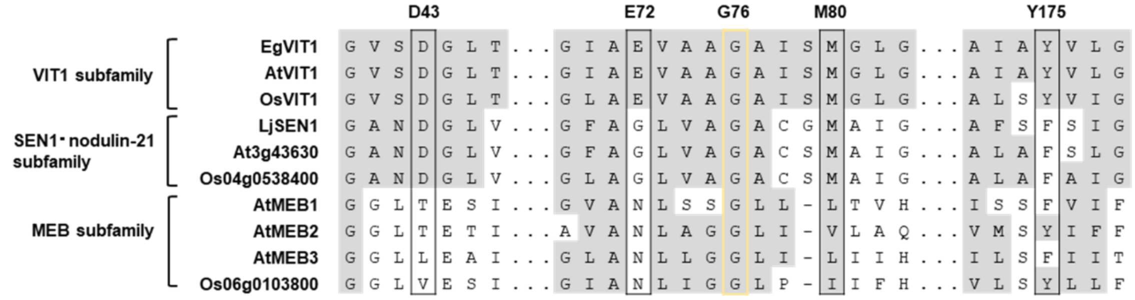

Supplementary Figure S1. Aliment of DUF125 domains, including key amino acid residues required for iron transport activity. Black open boxes indicate amino acid residues important for the iron transport activity of EgVIT1 (i.e., D43, E72, M80, and Y175) (Kato et al. 2019). The orange-colored open box indicates the Gly76 residue of EgVIT1, which is crucial for iron transport activity (Mary et al. 2015). The proteins used in the analysis are listed in Supplementary Table S1. '-' indicates deleted amino acids.

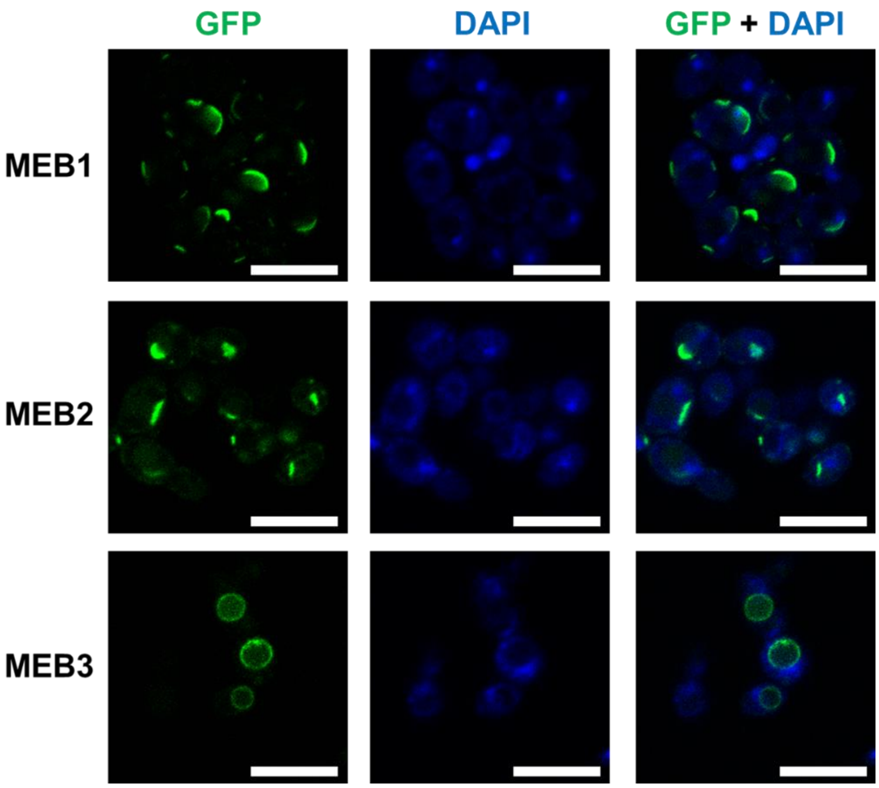

Supplementary Figure S2. Subcellular localization assays of GFP-MEB1, GFP-MEB2, and GFP-MEB3 fusion proteins in yeast cells. The nucleus was stained with the fluorescent dye, DAPI. Scale bars = 10 μm.

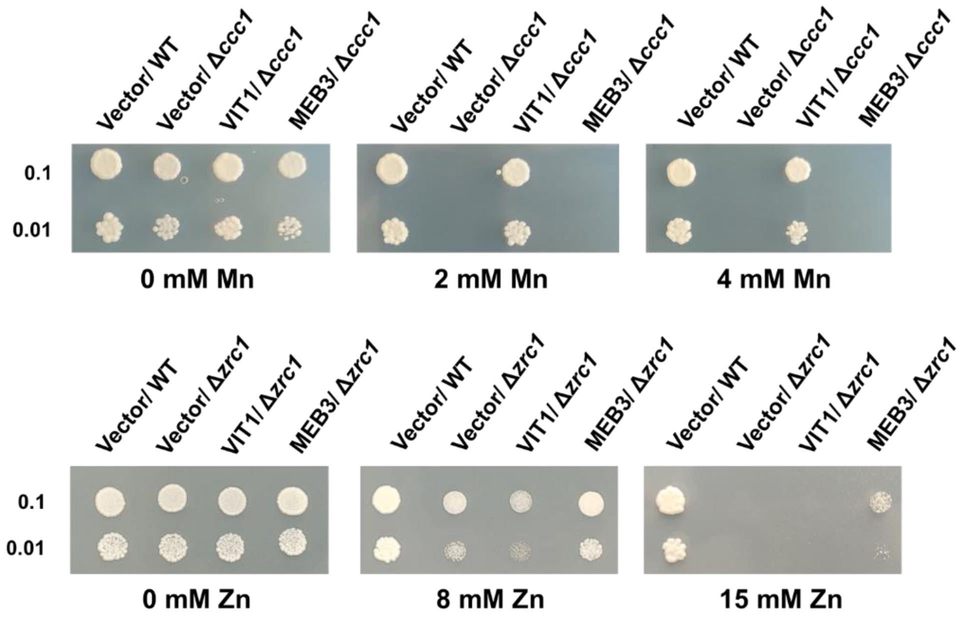

Supplementary Figure S3. Functional complementation assay of MEB3 using zinc and manganese sensitive yeast cells. The iron and manganese-sensitive strains (*ccc1*) or zinc-sensitive strain (*zrc1*) were grown in the SGal medium supplemented with or without manganese ($MnCl_2$) or zinc ($ZnSO_4$).

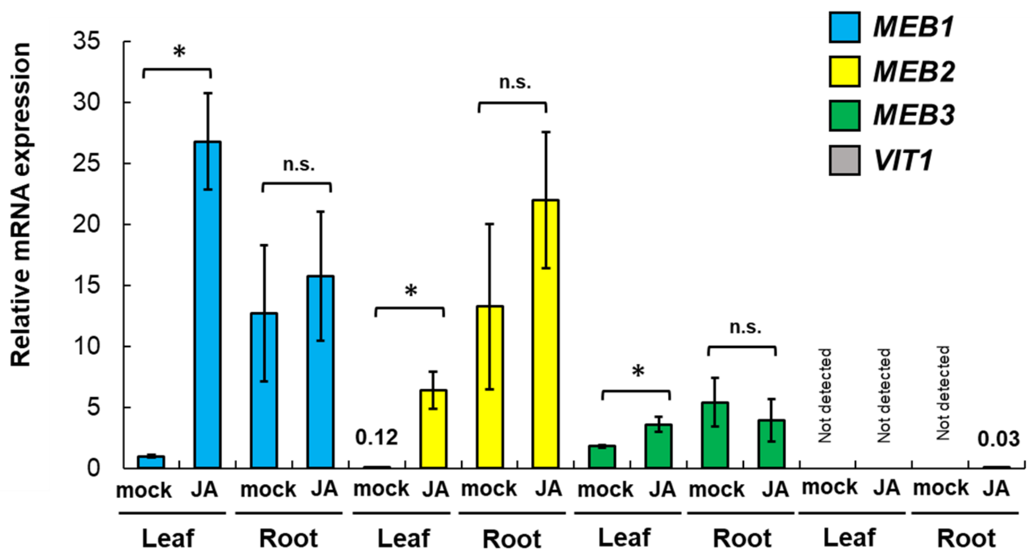

Supplementary Figure S4. Expression of *MEB1*, *MEB2*, *MEB3*, and *VIT1* in response to jasmonic acid (JA) treatment. The chart shows relative mRNA expression (*MEB1* expression in the leaf mock treatment was set as 1.0) of rosette leaves and roots incubated with 50 µM JA for 1 day. Data represent mean ± SE with biological replicates ($n = 4$). Asterisks above the columns indicate significant differences based on Student's *t*-test ($p < 0.05$). 'n.s.' indicates not significant.

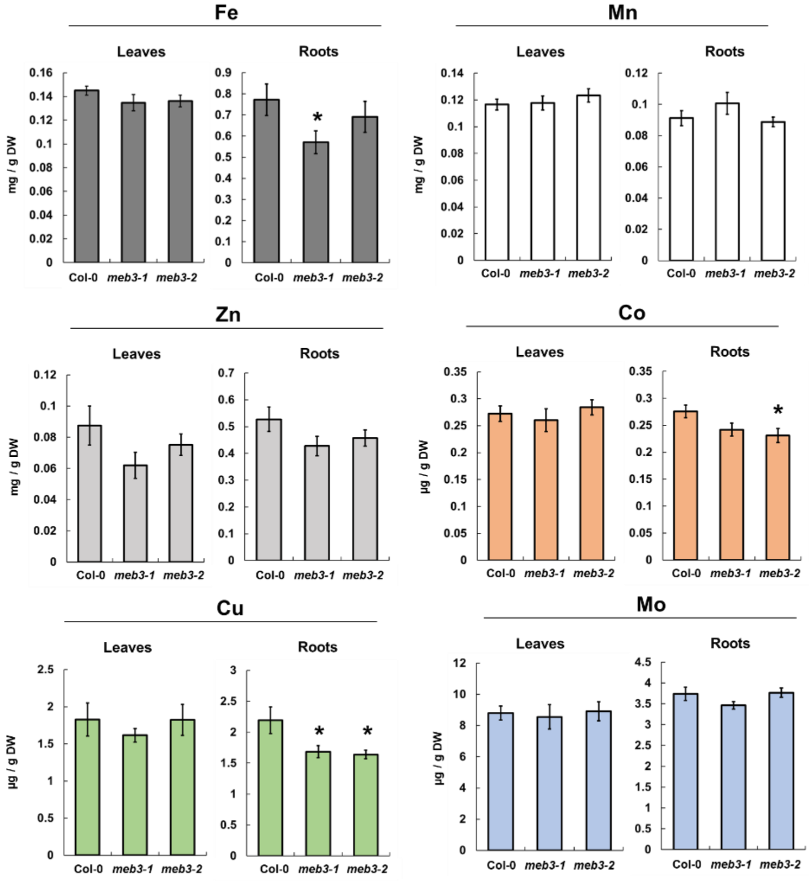

Supplementary Figure S5. Iron, manganese, zinc, molybdate, cobalt, and copper contents of the leaves and roots of 14-day-old plants grown on 1/2 MS medium containing 50 µM Fe. Plants used in this experiment are the same as those shown in Figure 6C experiment. The metals were included in MS medium as plant nutrients. Data represent mean ± SE with biological replicates (*n* = 10). Asterisks above the columns indicate significant differences with respect to Col-0 based on Student's *t*-test ($p < 0.05$).

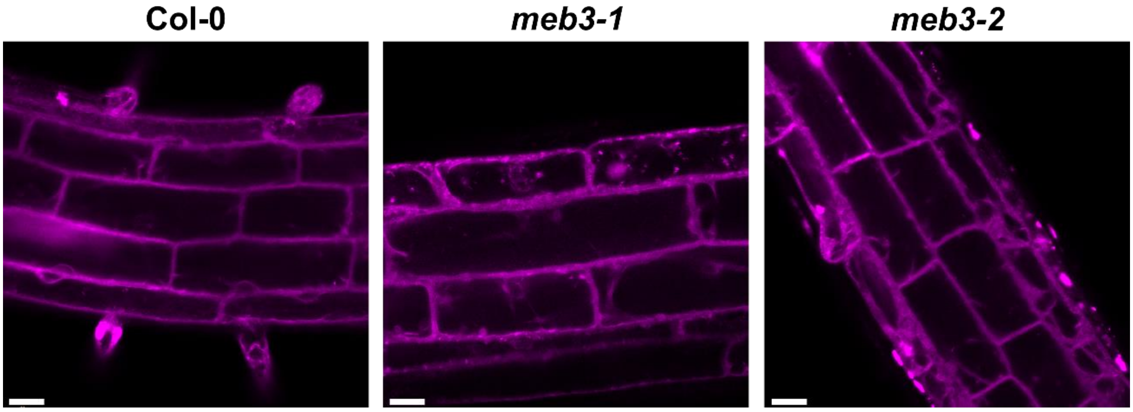

Supplementary Figure S6. Visualization of the root vacuolar membrane of 7-day-old Col-0, *meb3-1*, and *meb3-2* seedlings grown on 1/2 MS medium. The seedlings were stained by 4 µM FM4-64 vital dye for 30 min and then incubated in 1/2 MS medium for 3 hours. Scale bars = 20 µm.

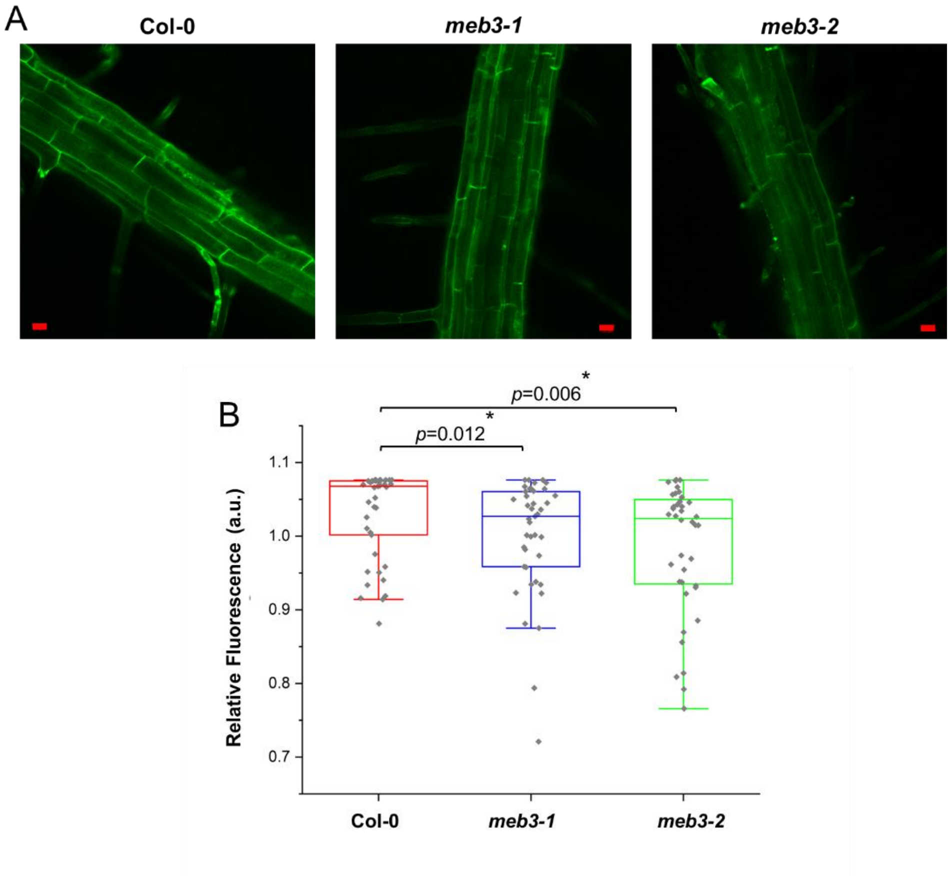

Supplementary Figure S7. Detection of labile Fe^{3+} using MPNBD fluorescent probes in roots. 7-day-old Col-0, *meb3-1*, and *meb3-2* seedlings were grown on 1/2 MS medium containing 50 µM Fe. A, An image showing Fe^{3+} fluorescence signals in roots. Scale bars = 20 µm. B, Quantification of the Fe^{3+} signal inside root cells. The fluorescence intensity inside cells was calculated by ImageJ software. Data are representative of three independent plants (n = 40 - 41 root cells). Asterisks above the columns indicate significant differences based on a non-parametric Wilcoxon Signed Rank test. ($p < 0.05$). a.u.= arbitrary unit.

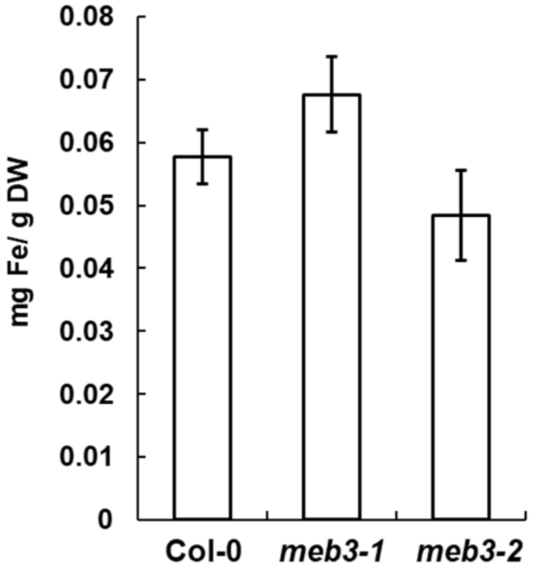

Supplementary Figure S8. Magnified chart of root iron contents of plants grown on no-iron medium, as shown in Figure 6D. Data represent mean ± SE with biological replicates (n = 3).

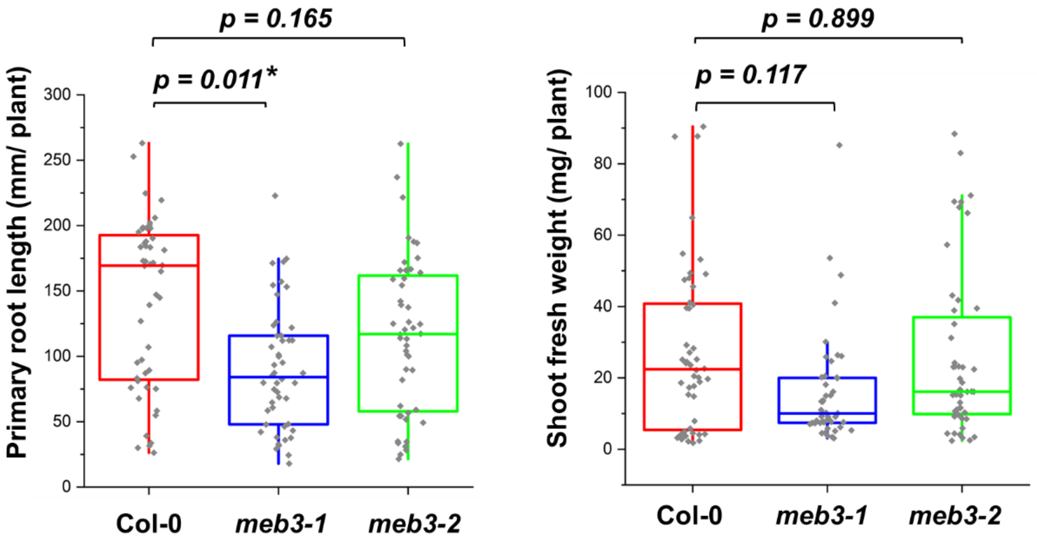

Supplementary Figure S9. Primary root length and shoot fresh weight of Col-0, *meb3-1*, and *meb3-2* plants grown in iron excess medium. Plants were germinated in normal medium (50 µM iron) for 5 days and then transferred to iron excess (+Fe, 200 µM iron) medium for 16 days. Asterisks indicate significant differences between Col-0 and mutant plants. Data are representative of three biological experiments. The *p*-values displayed on the boxplot were obtained using one-way ANOVA with a Tukey's HSD test applied over log2-transformed data.

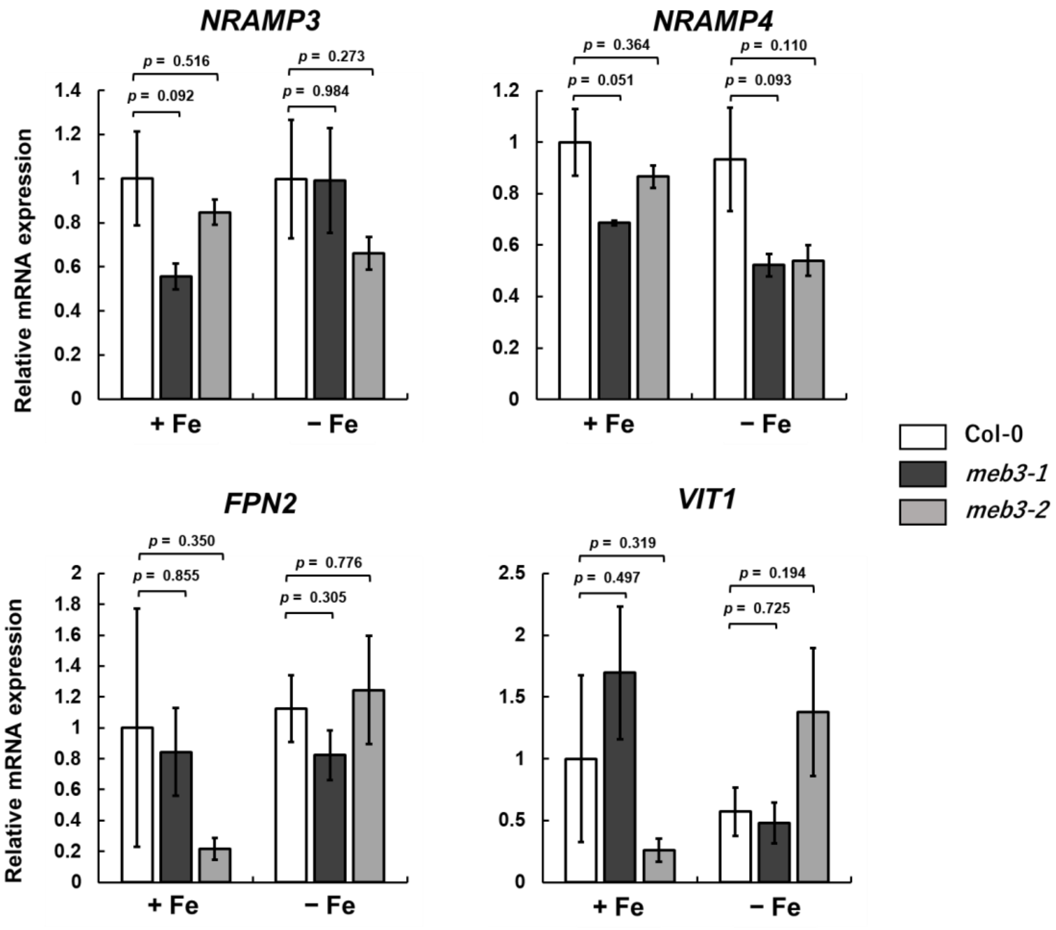

Supplementary Figure S10. Expression of vacuolar iron transporter genes in the roots of the *meb3* mutant. The cDNA used in this experiment is the same as that used in the experiment in Figure 8A. The charts show the relative mRNA expression of each gene (expression in Col-0 +Fe was set as 1.0). The error bars indicate SE with biological replicates (n = 4). The *p*-value between Col-o and mutants under each iron condition was calculated based on a Student's *t*-test.

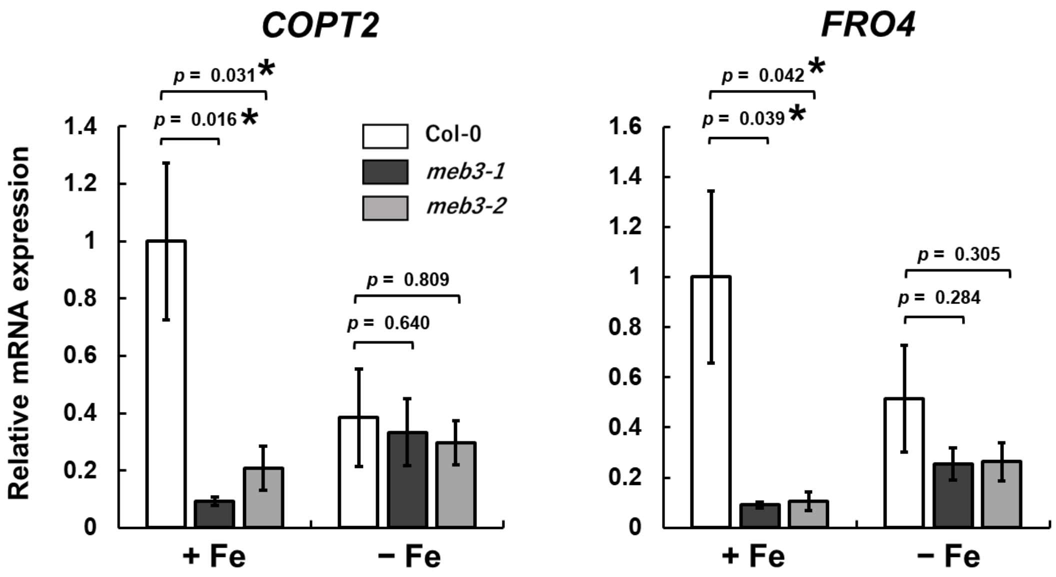

Supplementary Figure S11. Expression of copper uptake genes in the roots of the *meb3* mutant. The cDNA used in this experiment is the same as that used in the experiment in Figure 8A. The charts show the relative mRNA expression of each gene (expression in Col-0 +Fe was set as 1.0). The error bars indicate SE with biological replicates ($n = 4$). The *p*-value between Col-o and mutants under each iron condition was calculated based on a Student's *t*-test.

Table S1 List of proteins used in phylogenetic tree analysis in Fig. 1.

UniProt ID *	Gene name	Common name	Species	Protein subfamily **
Q9ZUA5	At2g01770	AtVIT1/VIT1	*Arabidopsis thaliana*	VIT1 subfamily
Q8W4P8	At4g27860	AtMEB1/MEB1	*Arabidopsis thaliana*	MEB subfamily
F4KFS7	At5g24290	AtMEB2/MEB2	*Arabidopsis thaliana*	MEB subfamily
Q8LPT3	At4g27870	AtMEB3/MEB3	*Arabidopsis thaliana*	MEB subfamily
Q9LPU9	At1g21140	AtVTL1/VTL1	*Arabidopsis thaliana*	VTL subfamily
Q9SRD3	At1g76800	AtVTL2/VTL2	*Arabidopsis thaliana*	VTL subfamily
Q9M2C3	At3g43630	AtVTL3/VTL3	*Arabidopsis thaliana*	VTL subfamily
Q9M2C0	At3g43660	AtVTL4/VTL4	*Arabidopsis thaliana*	VTL subfamily
Q9LSF6	At3g25190	AtVTL5/VTL5	*Arabidopsis thaliana*	VTL subfamily
A0A078FBU7	BnaA02g25920D		*Brassica napus*	VIT1 subfamily
A0A078JDR3	BnaC02g47710D		*Brassica napus*	VIT1 subfamily
A0A078FY03	BnaA06g34610D		*Brassica napus*	VIT1 subfamily
A0A078FXC5	BnaC01g20480D		*Brassica napus*	MEB subfamily
-	BnaA01g16700D		*Brassica napus*	MEB subfamily
-	BnaC07g30170D	BnMEB2	*Brassica napus*	MEB subfamily
-	BnaA06g26800D		*Brassica napus*	MEB subfamily
-	BnaA01g16710D		*Brassica napus*	MEB subfamily
A0A078G0N9	BnaC01g20490D		*Brassica napus*	MEB subfamily
G7LBG9	Mtr8g105790		*Medicago truncatula*	VIT1 subfamily
G7LBH0	Mtr8g105810		*Medicago truncatula*	VIT1 subfamily
G7K650	Mtr5g068580		*Medicago truncatula*	MEB subfamily
A0A072TKY1	Mtr8g012900		*Medicago truncatula*	MEB subfamily
A0A072U844	Mtr6g034975		*Medicago truncatula*	VTL subfamily
G7ID02	Mtr1g099010		*Medicago truncatula*	VTL subfamily
G7IL91	Mtr2g008110		*Medicago truncatula*	VTL subfamily
A0A072UNI3	Mtr4g094335	MtVTL8	*Medicago truncatula*	VTL subfamily
A0A072UQC2	Mtr4g094325	MtVTL4	*Medicago truncatula*	VTL subfamily
A0A072UPG2	Mtr4g094332		*Medicago truncatula*	VTL subfamily
A0A072UZH2	Mtr4g094330		*Medicago truncatula*	VTL subfamily
P16313		nodulin-21/ GmVTL1a	*Glycine max*	VTL subfamily
H3JSR8		LjSEN1/SEN1	*Lotus japonicus*	VTL subfamily
P0DO17		EgVIT1	*Eucalyptus grandis*	VIT1 subfamily
A0A3Q7FXW3	Solyc04g008060		*Solanum lycopersicum*	VIT1 subfamily
A0A3Q7FMX6	Solyc03g096800		*Solanum lycopersicum*	MEB subfamily
A0A3Q7F0P5	Solyc02g068260		*Solanum lycopersicum*	VTL subfamily
A0A3Q7EQK4	Solyc01g104780		*Solanum lycopersicum*	VTL subfamily
Q6MWE5	Os04g0463400	OsVIT1	*Oryza sativa*	VIT1 subfamily
Q6ERE5	Os09g0396900	OsVIT2	*Oryza sativa*	VIT1 subfamily
A0A0P0WRU6	Os06g0103800		*Oryza sativa*	MEB subfamily
Q6H658	Os02g0644200		*Oryza sativa*	VTL subfamily
B7F138	Os04g0538400		*Oryza sativa*	VTL subfamily
Q7XTL7	Os04g0686800		*Oryza sativa*	VTL subfamily
W1PVP7	AMTR_s00176p00023280		*Amborella trichopoda*	VIT1 subfamily
W1PU29	AMTR_s00027p00225770		*Amborella trichopoda*	MEB subfamily
U5DB74	AMTR_s00059p00177640		*Amborella trichopoda*	VTL subfamily
W1NST5	AMTR_s01243p00009970		*Amborella trichopoda*	VTL subfamily
A9SGF0	Pp3c2_34540		*Physcomitrium patens*	VIT1 subfamily
A0A2K1J7J0	Pp3c16_7270		*Physcomitrium patens*	(VIT1 subfamily like)
A0A2K1ID69	Pp3c25_40		*Physcomitrium patens*	(VIT1 subfamily like)
A0A2K1IVL0	Pp3c20_17480		*Physcomitrium patens*	(VTL subfamily like)
A0A2K1JWD5	Pp3c11_26180		*Physcomitrium patens*	VTL subfamily
A9T1R5	Pp3c20_1770		*Physcomitrium patens*	VTL subfamily
P47818	YLR220W	ScCCC1/CCC1	*Saccharomyces cerevisiae*	
Q9P6J2	SPBC1683.10c	SpPCL1	*Schizosaccharomyces pombe*	
Q97TY3	SULZ_RS05215		*Saccharolobus solfataricus*	
Q9WYW7	TM_0497		*Thermotoga maritima*	
Q9HXF5	PA3851		*Pseudomonas aeruginosa*	

* The UniProt database is https://www.uniprot.org. ** Deta are shown in Fig. 1.

Table S2 Summary statistics for the mesurements shoot fresh weight and primary root length in Fig. 7.

Genotype	Condition	Number of plants	Mean	Median	S.D.	Plant trait (unit)
Col-0	+ Fe	40	161.6	127.2	96.9	Shoot fresh weight (mg/ plant)
Col-0	− Fe	39	49.9	46.8	27.4	Shoot fresh weight (mg/ plant)
meb3-1	+ Fe	41	148.1	136.7	83.8	Shoot fresh weight (mg/ plant)
meb3-1	− Fe	49	42.7	34.8	31.3	Shoot fresh weight (mg/ plant)
meb3-2	+ Fe	38	159.0	157.4	75.6	Shoot fresh weight (mg/ plant)
meb3-2	− Fe	48	35.2	34.9	24.0	Shoot fresh weight (mg/ plant)
Col-0	+ Fe	55	87.2	92.6	33.6	Primary root length (mm/ plant)
Col-0	− Fe	46	65.9	71.8	25.6	Primary root length (mm/ plant)
meb3-1	+ Fe	58	84.5	87.0	15.1	Primary root length (mm/ plant)
meb3-1	− Fe	53	58.5	52.5	24.3	Primary root length (mm/ plant)
meb3-2	+ Fe	69	76.3	81.3	27.9	Primary root length (mm/ plant)
meb3-2	− Fe	58	52.7	50.6	26.5	Primary root length (mm/ plant)

Table S3 List of primers used in this study

Primer	Sequencce (5' > 3')
**qRT-PCR**	
RT-MEB1_F	TAGTGTTGAACCACGCAAAGGC
RT-MEB1_R	TGTTACCGTGCATAGGCTTGTG
RT-MEB2_F	CCGAGAATTTCGATCCAAGG
RT-MEB2_R	TTTGAACCGGTTTCGCTCAC
RT-MEB3_F	TGCTTGGCGGGCTTATTC
RT-MEB3_R	ACCGACCTTCTTCTTCTTCTCTACC
RT-VIT1_F	ATCGAACCGGAGAAGCAAAC
RT-VIT1_R	AGAGACGCCGATGATGATGTC
RT-IRT1_F	GCGTTCTTTTTCGCGGTAAC
RT-IRT1_R	GCGGAGCATGCATTTAGAAG
RT-UBQ10_F	GAAGTGGAAAGCTCCGACAC
RT-UBQ10_R	TTAGAAACCACCACGAAGACG
RT-FRO2_F	GTGCAATTCTCGCATCTTCG
RT-FRO2_R	TGTGGTCGATCGGGTAAATG
RT-FIT_F	AAGGAGAAGGTGTTGCTCCATC
RT-FIT_R	TGTTCGAAGCATGTCCCATC
RT-bHLH38_F	AGCAGCAACCAAAGGCG
RT-bHLH38_R	CCACTTGAAGATGCAAAGTGTAG
RT-bHLH100_F	AAGTCAGAGGAAGGGGTTACA
RT-bHLH100_R	GATGCATAGAGTAAAAGAGTCGCT
RT-AHA2_F	TTGAACGTCCTGGAGCATTG
RT-AHA2_R	AGTTGGCGTAAACCGCAATC
RT-NRAMP3_F	TCGATGTCTTAAACGAGTGGCTT
RT-NRAMP3_R	CGCGATTGTCTTGTACAAAGGA
RT-NRAMP4_F	TTGCTTGGTCTCCAATGAGC
RT-NRAMP4_R	CAAATTCGTTGCAGCTCCTG
RT-FPN2_F	TTTTCGGTCCCATTGTAGGC
RT-FPN2_R	ACCACCAGCAACAATGAAGG

RT-COPT2_F	CTGGCCTTTCGTATTTGGTG
RT-COPT2_R	AAGAAACCAACGCCATAGCC
RT-FRO4_F	TCACGGTTCCCATGATGTTC
RT-FRO4_R	ATCCCCAATGGGTTCATCAC
**Cloning**	
MEB3-DTFW	CACCATGGGATCCGCCGCCGA
MEB3-RV	TCAGTACGATGATGAGTATCC
pENTR_VIT1_F	GGAACCAATTCAGTCGACATGTCGTCGGAGGAAGATAAG
pENTR_VIT1_R	GAAAGCTGGGTCTAGATCTAATGTTGCACAACTTTAGCC
pBI121MEB3_F	CATGATTACGCCAAGCTTCATTTTAATCATGGATCAAATTCAG
pBI121MEB3_R	CTGACCACCCGGGGATCCCAGAAAGGGAAAAGATGACAAAG
